# Are estimates of socioeconomic inequalities in chronic disease artefactually narrowed by self-reported measures of prevalence in low-income and middle-income countries? Findings from the WHO-SAGE survey

**DOI:** 10.1136/jech-2014-204621

**Published:** 2014-12-30

**Authors:** Sukumar Vellakkal, Christopher Millett, Sanjay Basu, Zaky Khan, Amina Aitsi-Selmi, David Stuckler, Shah Ebrahim

**Affiliations:** 1 Public Health Foundation of India, New Delhi, India; 2 Department of Sociology, Oxford University, Oxford, UK; 3 Department of Primary Care and Public Health, Imperial College London, London, UK; 4 Prevention Research Center, Stanford University, Stanford, Palo Alto, California, USA; 5 Department of Public Health and Policy, London School of Hygiene and Tropical Medicine, London, UK; 6 Department of Epidemiology & Public Health, University College London, UK; 7 Department of Non-Communicable Disease Epidemiology, London School of Hygiene and Tropical Medicine, London, UK

**Keywords:** Epidemiology of chronic non communicable diseases, INEQUALITIES, PUBLIC HEALTH

## Abstract

**Background:**

The use of self-reported measures of chronic disease may substantially underestimate prevalence in low-income and middle-income country settings, especially in groups with lower socioeconomic status (SES). We sought to determine whether socioeconomic inequalities in the prevalence of non-communicable chronic diseases (NCDs) differ if estimated by using symptom-based or criterion-based measures compared with self-reported physician diagnoses.

**Methods:**

Using population-representative data sets of the WHO Study of Global Ageing and Adult Health (SAGE), 2007–2010 (n=42 464), we calculated wealth-related and education-related concentration indices of self-reported diagnoses and symptom-based measures of angina, hypertension, asthma/chronic lung disease, visual impairment and depression in three ‘low-income and lower middle-income countries’—China, Ghana and India—and three ‘upper-middle-income countries’—Mexico, Russia and South Africa.

**Results:**

SES gradients in NCD prevalence tended to be positive for self-reported diagnoses compared with symptom-based/criterion-based measures. In China, Ghana and India, SES gradients were positive for hypertension, angina, visual impairment and depression when using self-reported diagnoses, but were attenuated or became negative when using symptom-based/criterion-based measures. In Mexico, Russia and South Africa, this distinction was not observed consistently. For example, concentration index of self-reported versus symptom-based angina were: in China: 0.07 vs −0.11, Ghana: 0.04 vs −0.21, India: 0.02 vs −0.16, Mexico: 0.19 vs −0.22, Russia: −0.01 vs −0.02 and South Africa: 0.37 vs 0.02.

**Conclusions:**

Socioeconomic inequalities in NCD prevalence tend to be artefactually positive when using self-report compared with symptom-based or criterion-based diagnostic criteria, with greater bias occurring in low-income countries. Using standardised, symptom-based measures would provide more valid estimates of NCD inequalities.

## Introduction

A significant limitation to the currently available evidence on the magnitude and direction of socioeconomic inequalities in non-communicable chronic diseases (NCDs) is the widespread use of self-reported cases of physician diagnoses as source data. These self-reported data may systematically skew estimated inequalities due to reporting bias. Wealthier and more educated individuals tend to have relatively better access to healthcare as well as greater knowledge about disease conditions, compared with those in lower socioeconomic status (SES) groups.^1–4^ Since the governance of health systems and public service infrastructure in lowand middle income countries (LMICs) are generally weak, the gap in access to healthcare between low and high SES individuals is greater in lower income countries compared to higher income countries.^5^ As a result, the use of self-reported measures of NCDs may systematically underestimate the true NCD prevalence and could lead to an artificial inequality that makes social gradients appear positive, especially in LMICs.^6–8^

The use of standardised measures of identification of disease (ie, case finding) that assume no prior diagnosis or patient knowledge can help minimise reporting bias.^7^ ^9^ The use of more objective measures consisting of symptom-based and criterion-based measures of diseases from the population surveys can be a viable option. For example, the symptom-based measure of angina using the WHO-Rose angina questionnaire^10^ has been widely used in epidemiological studies.^11^ ^12^ Furthermore, symptom-based prevalence measures of asthma^13^ and depression^14^ were used in studies in different cultural settings. Several household level surveys in LMICs collect detailed disease specific information of symptoms and anthropometric features which can generate standardised disease measures.^15^ ^16^ Several studies have incorporated more criterion-based measures of prevalence of specific NCDs in LMICs,^6^ ^8^ ^17–20^ and shown higher prevalence rates and negative SES gradients in NCDs; however, little evidence is available on whether prevalence rates and SES inequalities of specific NCDs vary when standardised measures and self-reported diagnoses are employed and the extent to which these vary in countries at different stages of economic development.

In this study, we tested whether and to what extent SES inequalities in the prevalence of NCDs were less marked if estimated using symptom-based or criterion-based measures, which assume no prior diagnosis or patient knowledge, compared with self-reported physician diagnoses. We evaluated the prevalence of five NCDs (angina, hypertension, asthma/chronic lung diseases, visual impairment and depression) in six LMICs. On the basis of the World Bank classification of countries, we grouped China, Ghana and India into ‘low-income and lower middle-income countries’ and Mexico, Russia and South Africa into ‘upper-middle-income countries’. We hypothesise that (1) SES gradients in NCD prevalence would be more likely to be positive if measured by self-reported physician diagnoses than by symptom-based/criterion-based measures, and that (2) these estimated SES inequalities would be greater in China, Ghana and India than in Mexico, Russia and South Africa. We discuss the findings in the light of the observation that lower SES groups in lower-income countries are at a greater disadvantage from their poorer knowledge of NCDs and lower access to healthcare than in richer countries as described above.

## Methods

We used the individual level, cross-sectional data of wave 1 from the WHO Study on Global AGEing and Adult Health (SAGE), covering the years 2007–2010. SAGE is a series of nationally representative samples of persons aged 50+years and younger adults aged 18–49 years in China, Ghana, India, Mexico, Russia and South Africa.^21^ These SAGE countries represent different geographic regions of the world, levels of economic development and stages in the demographic and health transition, including the world's two most populous countries, China and India.^21^ Comparative descriptions of the six countries are presented in web appendix table 1.

The sampling method used for SAGE was based on the design for the World Health Survey 2002–2004, which was drawn from the national census of each country. The sampling details of SAGE have been documented elsewhere.^15^ ^21^ Briefly, SAGE employed a probability sampling strategy using multistage, stratified, random cluster samples. The primary sampling units were stratified by region and location (urban/rural), and enumeration areas were selected within each stratum. The samples were drawn from a national sampling frame using a stratified, multistage cluster design so as to allow each household and individual respondent to be assigned a known non-zero probability of selection. The households were classified into one of two mutually exclusive categories:^1^ ‘50+ household’, and^2^ ‘18–49 household’. In the sample of age 50+ households, all individuals aged 50 years or older were eligible for interview and invited to respond. Only one individual aged 18–49 years was selected from the sample of age 18–49 households, and this individual was randomly selected using Kish grid table, so to avoid skewing the sample towards particular age or sex groups. Household-level and person-level analysis weights were calculated for each country, which included sample selection and a post-stratification factor.^21^

Standardised survey instruments, interviewer training and translation protocols were used in all SAGE countries. Interviewer-administered questionnaires in the native language of the respondent using local, commonly understood terms, with back translation to English to ensure accuracy and comparability, were used. Interviews were conducted between 2007 and 2010. The pooled wave 1 six-country totals for individual respondents included 34 124 respondents aged 50 years and 8340 aged 18–49 years. The individual level response rates for each country were as follows: China (93%), Ghana (81%), India (68%), Mexico (53%), Russia (83%) and South Africa (75%). SAGE achieved a very high response rate for two reasons. First, the majority of the respondents were participants in the World Health Survey 2002–2004 and followed up. Second, local institutions applied concerted efforts in collaboration with local partners to improve survey response. This included conducting a minimum of three revisits to households.

Ethical clearance was obtained from local research review boards for each participating SAGE site, in addition to the WHO Ethical Review Committee. Informed consent was obtained from each respondent prior to interview.

### Measuring NCDs prevalence

Of the total eight NCDs reported in the SAGE survey, we considered five major NCDs: angina, hypertension, chronic lung diseases (emphysema, bronchitis, chronic obstructive pulmonary disease (COPD)) and asthma, visual impairment and depression. We excluded diabetes, stroke and arthritis because data were insufficient or unavailable for developing symptom-based measures.

The descriptions of survey questions for self-reported physician diagnoses and the criteria used for deriving symptom-based measures for each NCD were presented in web appendix table 2.

We derived symptom-based/criterion-based measures from the literature and previous household surveys. Symptom-based prevalence measures, which were based on the respondent's self-report, were used for angina and depression. Objective measures of prevalence based on internationally accepted standard criteria were employed for hypertension, asthma and chronic lung diseases, and visual impairment. The symptom-based measure for angina was from the WHO-Rose angina questionnaire,^10^ which has been widely used in epidemiological studies.^11^ ^12^ We used the cut-offs for high blood pressure based on systolic blood pressure ≥140 mm Hg and/or diastolic blood pressure ≥90 mm Hg, the WHO criteria for diagnosing hypertension in adults 18 years and older.^22^ ^23^ Symptom-based measures are available for asthma^13^ but not for chronic lung diseases. Hence, we used the spirometry test as a criterion-based measure for asthma and chronic lung diseases, as per the Global Initiative for Obstructive Lung Disease (GOLD) criteria for identifying obstructive diseases that would include asthma, COPD, chronic bronchitis and emphysema.^24^ The prevalence of visual impairment was estimated using the Tumbling E LogMAR chart.^25^ ^26^ Finally, criteria for ‘moderate depression’ were derived from the International Statistical Classification of Diseases (ICD)-10 classification of mental and behavioural disorders.^27^ The Center for Epidemiologic Studies Depression Scale Revised (CESD-R), developed by the Center for Epidemiologic Studies, has been widely used to measure depression in a different cultural setting.^14^ We used the ICD-10 criteria of depression for the symptom-based measure as SAGE had used the same criteria of depression.

### Socioeconomic status

We used wealth (asset-score index) and education as two distinct indicators of SES. A validated asset (wealth) score index, as originally reported in the WHO SAGE data set, was derived using the WHO standard approach to estimate permanent income from survey data on household ownership of durable goods, neighbourhood and dwelling characteristics, and access to water, sanitation and electricity.^28^ For the bivariate tabulation, education was defined as five categories such as ‘No formal education’, ‘Less than primary school’, ‘Primary school completed’, ‘High/secondary school’, and ‘College/university education’. For estimating the multivariate logistic regression and concentration index, we used the years of education.

### Modelling approach

We estimated wealth-based and education-based concentration indices to estimate the SES inequalities in NCDs. The concentration index has been increasingly used to measure socioeconomic inequalities in health in an objective and readily understandable manner.^29–31^ The concentration index, a generalisation of the Gini coefficient, takes the whole socioeconomic distribution of the population into account, and is mathematically equivalent to the slope and relative index of inequality. The Concentration index (C) was computed as twice the (weighted) covariance of the health variable (‘ill-health’ in the present study) and a person's relative rank in terms of economic status, divided by the variable mean, according to the equation below.1
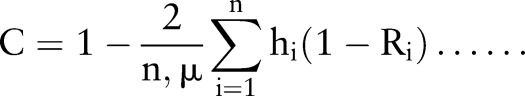
where n is the sample size, h_i_ is the ill health of the ith individual, µ is the weighted mean of the ill-health, R_i_ is the fractional rank of the ith individual in terms of the index of household economic status. The concentration index can vary between −1 (concentrated among lower SES) and +1 (concentrated among the higher SES), and zero when there is no inequality.^32^ In addition, we estimated multivariate logistic regression models for each NCD.

We adjusted the estimates of concentration index and logistic regression models of symptom-based/criterion-based measures of NCD for the differential healthcare access across SES groups, which can potentially bias the symptom-based/criterion-based measures. For example, the acute pain symptom from angina is often amenable to medication (eg, sublingual nitroglycerine or removal of physical exertion), but otherwise would be largely asymptomatic if the patient is on chronic treatment. Access to these treatments might well be different for different SES respondents and might well account for the differences in the outcomes. In our models, we included a dichotomous variable of whether the respondent has taken medicines/treatments in the past 2 weeks for the criterion-based measure of hypertension, and asthma and chronic lung diseases, undergone surgery to correct visual impairment, and taken medicines/treatments in the past 12 months for the symptom-based angina and depression. Longer time windows for symptom-based angina and depression were used as assessment of these was based on a 12-month recall-period.

All statistical estimations were done with STATA V.13.1 (Stata Corp, College Station, Texas, USA).

## Results

Table 1 shows the characteristics of the study sample of each country. The proportion of women respondents across countries ranged from 49.1% (China) to 57.3% (Russia). The respondents were drawn largely from rural areas in China (51%), Ghana (54%) and India (74%) while rural respondents consisted of a smaller proportion of the sample in Mexico (22%), Russia (24%) and South Africa (31%).

**Table 1:** Socioeconomic characteristics of the sample population of China, Ghana, India, Mexico, Russia and South Africa (weighted sample)

	Ghana	India	China	South Africa	Mexico	Russia
Total (N)	5573	12 198	14 785	4227	2734	3418
Sex (%)						
Women	50.4	50.3	49.1	52.8	52.0	57.3
Men	49.6	49.7	50.9	47.2	48.0	42.7
Age group (%)						
18–34	23.8	35.8	15.6	34.6	40.0	14.9
35–49	50.5	39.0	58.4	41.3	33.5	24.0
50–59	10.3	12.3	11.7	12.0	12.7	26.9
60+	15.3	12.9	14.3	12.1	13.7	34.1
Education level (%)						
No formal schooling	29.0	35.1	7.9	8.6	0.0	0.4
Less than primary school	13.5	8.9	11.9	12.2	21.5	0.5
Primary school completed	19.9	16.7	17.1	16.0	27.2	3.4
Secondary high school completed	33.4	30.8	53.5	56.0	38.1	73.3
College completed or above	4.2	8.5	9.6	7.3	13.2	22.4
Location (%)						
Rural	54.0	74.3	51.3	30.7	22.2	24.4
Urban	46.0	25.7	48.7	69.3	77.8	75.6

Source: WHO Study on Global AGEing and Adult Health (SAGE) survey, 2007–2010.

### Prevalence rate differences

**Figure 1:**
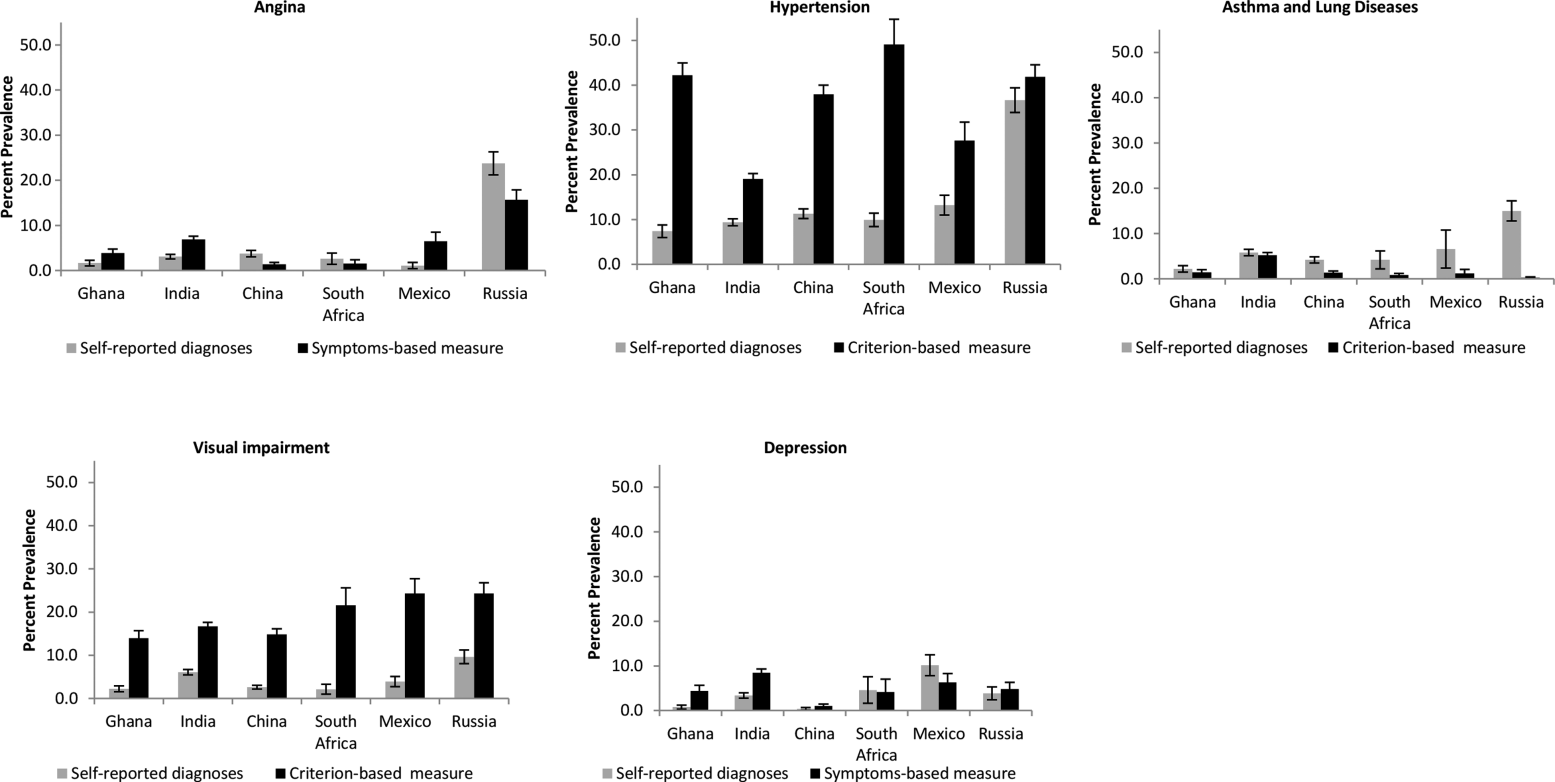
Self-reported diagnoses and symptoms-based/criterion-based measures of the prevalence rate (95% CI) of diseases among the adult population in China, Ghana, India, Mexico, Russia and South Africa.

Marked differences in the prevalence levels between self-reported physician diagnoses and symptom-based/criterion-based measures of NCDs were found between countries (figure 1). In China, Ghana and India, the prevalence of hypertension, visual impairment and depression was higher when symptom-based/criterion-based measures were used compared with self-reported diagnoses. The prevalence of symptom-based angina was also higher than that of self-reported angina in these countries, except in China. However, the prevalence of criterion-based measures of asthma and chronic lung disease was lower than that of self-reported diagnoses in these countries.

In Mexico, Russia and South Africa, we found a higher prevalence of hypertension and visual impairment using criterion-based measures, but a reverse trend was evident for asthma and chronic lung disease. In contrast, findings for angina and depression were heterogeneous, with the prevalence of symptom-based angina being lower in Russia and South Africa but higher in Mexico; also, the prevalence of symptom-based depression was lower in Mexico and South Africa, and higher in Russia.

### SES inequalities in NCDs

**Figure 2:**
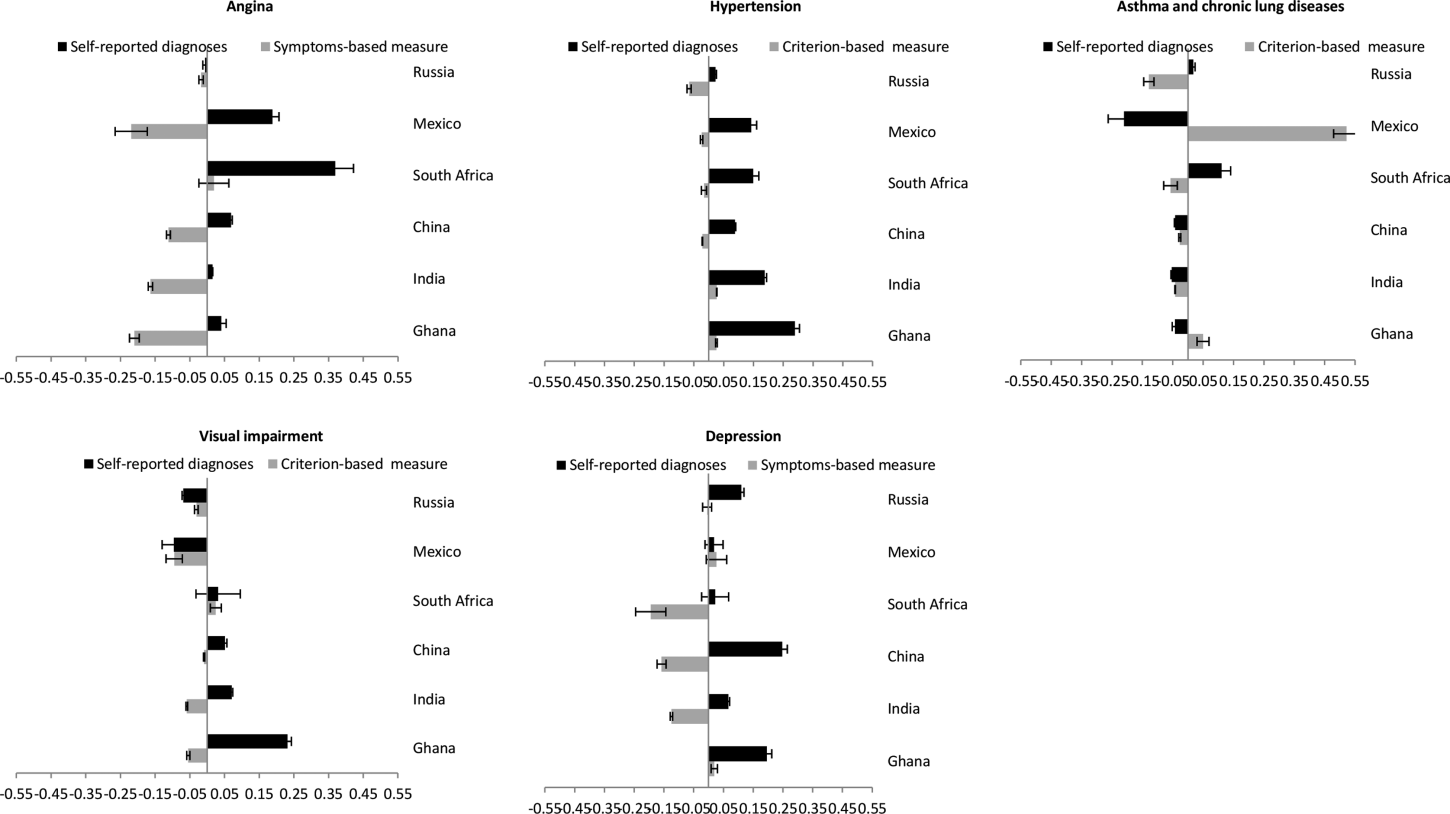
Age-standardised wealth-related and education-related concentration index (95% CI) of angina, hypertension, asthma and chronic lung diseases, visual impairment, and depression for self-reported diagnoses and symptoms-based/criterion-based measure, among the adult population in China, Ghana, India, Mexico, Russia and South Africa.

**Figure 3:**
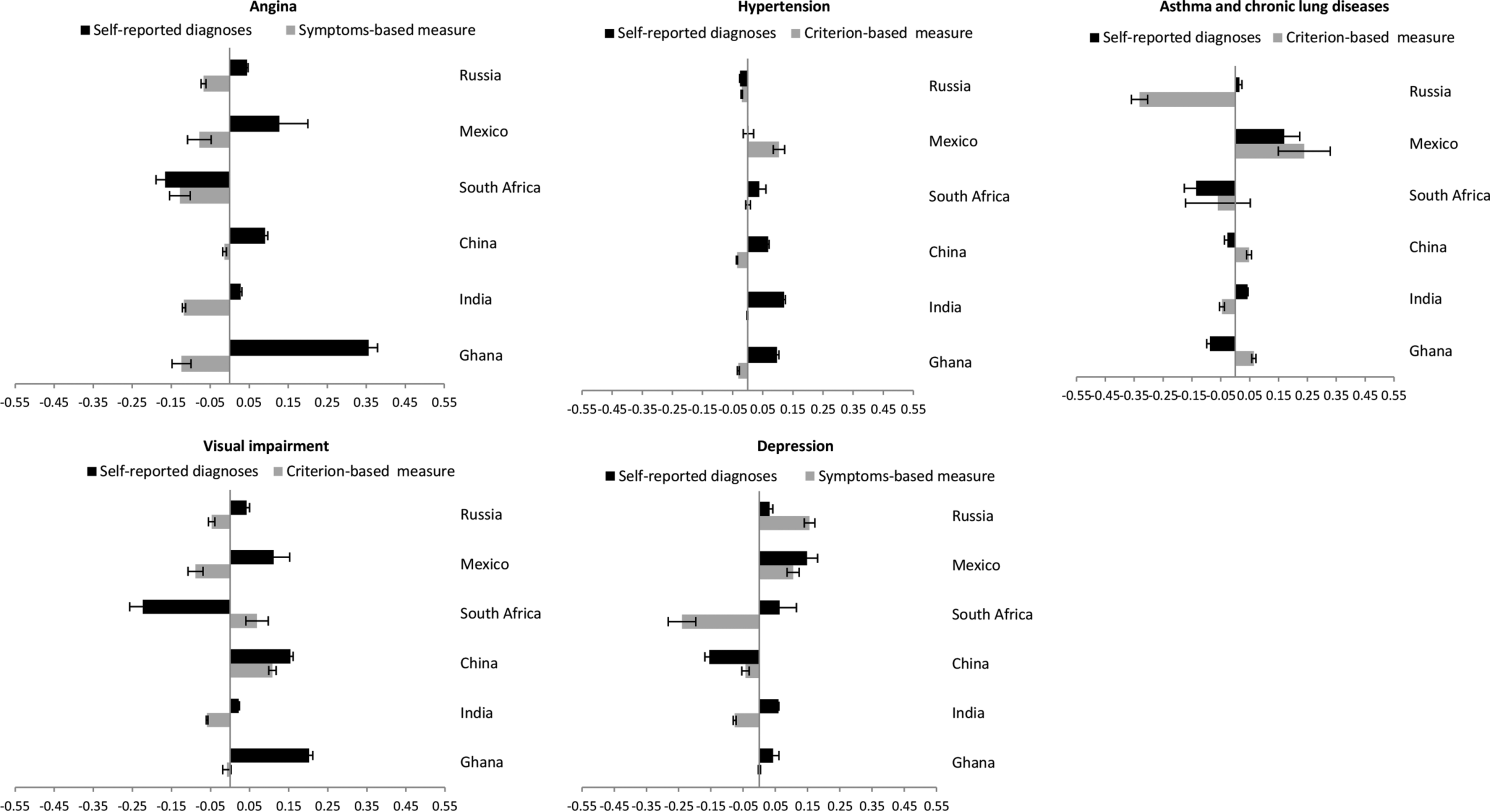
Age-standardised wealth-related and education-related concentration index (95% CI) of angina, hypertension, asthma and chronic lung diseases, visual impairment, and depression for self-reported diagnoses and symptoms-based/criterion-based measure, among the adult population in China, Ghana, India, Mexico, Russia and South Africa.

Within-country prevalence levels of symptom-based/criterion-based measures were higher among those with lower SES compared to self-reported diagnoses for most NCDs in China, Ghana and India (see web appendix tables 3 and 4, and web appendix figure 1). Figures 2A–E and 3 and table 2 show the concentration index of each NCD within country. Further, web appendix tables 5 and 6 show the adjusted and undjusted OR of gender, asset (wealth) and education on the prevalence of specific NCDs.

**Table 2:** Age-adjusted wealth-related and education-related concentration index (in %, with 95% CI) of disease, measured through self-reported diagnoses and symptoms-based/criterion-based measures, among the adult population in China, Ghana, India, Mexico, Russia and South Africa

	Wealth-related concentration index		Education-related concentration index	
	Symptoms-based/criterion-based measures	Self-reported diagnoses	Symptoms-based/criterion-based measures	Self-reported diagnoses
Angina				
Ghana	−0.21 (−0.22 to −0.20)	0.04 (0.03 to 0.05)	−0.12 (−0.15 to −0.10)	0.36 (0.33 to 0.38)
India	−0.16 (−0.17 to −0.16)	0.02 (0.01 to 0.02)	−0.12 (−0.12 to −0.11)	0.03 (0.03 to 0.03)
China	−0.11 (−0.12 to −0.11)	0.07 (0.07 to 0.07)	−0.01 (−0.02 to −0.01)	0.09 (0.08 to 0.10)
South Africa	0.02 (−0.02 to 0.06)	0.37 (0.32 to 0.42)	−0.13 (−0.15 to −0.10)	−0.17 (−0.19 to −0.14)
Mexico	−0.22 (−0.26 to −0.17)	0.19 (0.17 to 0.21)	−0.08 (−0.11 to −0.05)	0.13 (0.05 to 0.20)
Russia	−0.02 (−0.02 to −0.01)	−0.01 (−0.01 to 0.00)	−0.07 (−0.07 to −0.06)	0.04 (0.04 to 0.05)
Hypertension				
Ghana	0.03 (0.02 to 0.03)	0.29 (0.27 to 0.30)	−0.03 (−0.03 to −0.03)	0.10 (0.09 to 0.10)
India	0.03 (0.03 to 0.03)	0.19 (0.18 to 0.19)	0.00 (0.00 to 0.00)	0.12 (0.12 to 0.13)
China	−0.02 (−0.02 to −0.02)	0.09 (0.09 to 0.09)	−0.04 (−0.04 to −0.03)	0.07 (0.06 to 0.07)
South Africa	−0.02 (−0.03 to −0.01)	0.15 (0.13 to 0.17)	0.00 (−0.01 to 0.01)	0.04 (0.02 to 0.06)
Mexico	−0.02 (−0.03 to −0.02)	0.14 (0.12 to 0.16)	0.10 (0.08 to 0.12)	0.00 (−0.01 to 0.02)
Russia	−0.07 (−0.07 to −0.06)	0.02 (0.02 to 0.03)	−0.02 (−0.02 to −0.02)	−0.03 (−0.03 to −0.02)
Asthma and chronic lung disease				
Ghana	0.05 (0.03 to 0.07)	−0.04 (−0.05 to −.03)	0.06 (0.06 to 0.07)	−0.09 (−0.10 to −0.08)
India	−0.04 (−0.04 to −0.04)	−0.05 (−0.06 to −0.05)	−0.05 (−0.05 to −0.04)	0.04 (0.04 to 0.04)
China	−0.03 (−0.03 to −0.02)	−0.04 (−0.04 to−0.04)	0.05 (0.04 to 0.06)	−0.03 (−0.04 to −0.02)
South Africa	−0.06 (−0.08 to −0.03)	0.11 (0.08 to 0.14)	−0.06 (−0.17 to 0.05)	−0.14 (−0.18 to −0.09)
Mexico	0.52 (0.48 to 0.56)	−0.21 (−0.26 to −0.16)	0.24 (0.15 to 0.33)	0.17 (0.12 to 0.22)
Russia	−0.13 (−0.15 to −0.11)	0.02 (0.01 to 0.02)	−0.33 (−0.36 to −0.30)	0.01 (0.01 to 0.02)
Visual impairment				
Ghana	−0.05 (−0.06 to −0.05)	0.23 (0.22 to 0.24)	−0.01 (−0.02 to 0.00)	0.20 (0.19 to 0.21)
India	−0.06 (−0.06 to −0.06)	0.07 (0.07 to 0.07)	−0.06 (−0.06 to −0.06)	0.02 (0.02 to 0.02)
China	−0.01 (−0.01 to −0.01)	0.05 (0.05 to 0.06)	0.11 (0.10 to 0.12)	0.15 (0.15 to 0.16)
South Africa	0.02 (0.01 to 0.04)	0.03 (−0.03 to 0.10)	0.07 (0.04 to 0.10)	−0.22 (−0.26 to −0.19)
Mexico	−0.09 (−0.12 to −0.07)	−0.10 (−0.13 to −0.06)	−0.09 (−0.11 to −0.07)	0.11 (0.07 to 0.15)
Russia	−0.03 (−0.04 to −0.03)	−0.07 (−0.07 to −0.07)	−0.05 (−0.06 to −0.04)	0.04 (0.03 to 0.05)
Depression				
Ghana	0.02 (0.01 to 0.03)	0.20 (0.18 to 0.21)	0.00 (0.00 to 0.00)	0.04 (0.02 to 0.06)
India	−0.12 (−0.13 to −0.12)	0.07 (0.06 to 0.07)	−0.08 (−0.08 to −0.07)	−0.04 (−0.05 to −0.04)
China	−0.16 (−0.17 to −0.14)	0.25 (0.23 to 0.26)	−0.04 (−0.05 to −0.03)	−0.16 (−0.17 to−0.14)
South Africa	−0.19 (−0.25 to −0.14)	0.02 (−0.02 to 0.07)	−0.24 (−0.28 to −0.20)	0.06 (0.01 to 0.12)
Mexico	0.03 (−0.01 to 0.06)	0.02 (−0.01 to 0.05)	0.11 (0.09 to 0.12)	0.15 (0.12 to 0.18)
Russia	0.00 (−0.02 to 0.01)	0.11 (0.10 to 0.12)	0.16 (0.14 to 0.17)	0.03 (0.02 to 0.04)

(1) Source: WHO SAGE survey, 2007–2010; (2) Negative values indicate concentration of disease among those of lower socioeconomic status (SES), and positive values indicate concentration among those of higher SES The symptom-based/criterion-based measures of each non-communicable chronic disease was also adjusted with a dichotomous variable of whether the respondent has taken medication/treatment or not.

In China, Ghana and India, the prevalence of most NCDs was concentrated among higher SES groups based on self-reported diagnoses. Conversely, SES patterning was either attenuated or was concentrated in lower SES groups when based on symptom-based/criterion-based measures. Self-reported diagnoses (C_self-report_) for hypertension, angina, visual impairment and depression were more concentrated among higher SES individuals, whereas symptom-based/criterion-based measures (C_symptom or_ C_criterion-based_) of these NCDs showed either concentration among lower SES individuals or attenuation in the SES inequalities. For example, the age-standardised wealth-related concentration index of angina showed positive values (concentration among higher SES) for self-reported diagnoses and negative values (concentration among lower SES) for symptoms-based diagonoses (Ghana: C_self-report_ 0.04 vs C_symptom_ −0.21; India: C_self-report_ 0.02 vs C_symptom_ −0.16; China: C_self-report_ 0.07 vs C_symptom_ −0.11). Conversely, self-reported diagnoses of asthma and chronic lung diseases in China, Ghana and India were concentrated among lower SES individuals.

In Mexico, Russia and South Africa, the patterns of SES inequalities between self-reported diagnoses and symptom-based/criterion-based measures showed a mixed picture with some NCDs showing patterns similar to China, Ghana and India and others showing a heterogeneous pattern. More specifically, there was a higher prevalence among lower SES individuals of symptom-based/criterion-based measures of angina and hypertension than self-reported diagnoses. Self-reported hypertension was concentrated among higher SES individuals, whereas criterion-based measure of hypertension was concentrated among lower SES individuals (South Africa: C_self-report_ 0.15 vs C_criterion-based_ −0.02; Mexico: C_self-report_ 0.14 vs C_criterion-based_ −0.02; Russia: C_self-report_ 0.02 vs C_criterion-based_ −0.07)*.* Self-reported angina was concentrated among higher SES individuals in Mexico (C_self-report_ 0.19 vs C_symptom_ −0.22) and South Africa (C_self-report_ 0.37 vs C_symptom_ 0.02) while symptom-based angina was concentrated in lower SES individuals in Mexico but turned to get less positive in South Africa. Indicating similar patterns, symptom-based angina in Russia was more common among lower SES individuals. However, the SES concentration of most NCDs such as asthma and chronic lung diseases, visual impairment and depression in Mexico, Russia and South Africa showed more heterogeneous patterns. Web appendix table 7 summarises the main findings on the SES inequalities between self-reported diagnoses and symptom-based measures of specific NCDs across countries.

### Gender differences in prevalence and inequalities

Most NCDs were found to be more prevalent among women than men, irrespective of the method used for assessing prevalence (see web appendix table 8). For example, self-reported and symptom-based angina was more prevalent among women than men across all the six countries, with the exception of India, where a lower prevalence of self-reported angina was reported (3% in women vs 3.2% in men). Prevalence rates using self-reported and symptom-based/criterion-based measures in men and women also varied between NCDs. For example, the criterion-based measure of hypertension was more prevalent among men than women as compared with the self-reported measure in most countries, and a heterogeneous pattern for most of the other NCDs.

Patterns of SES inequality in NCDs also differed by sex and country (see web appendix table 9). In all countries except South Africa, the symptom-based measure of angina was more highly concentrated among lower SES men and women. Furthermore the criterion-based measure of visual impairment was amore among lower SES men and women in most countries, with the exception of higher prevalence among higher SES in China and South Africa. In contrast, the symptoms-based measure of depression was more among lower SES women across countries (except Mexico and Russia) and more among higher SES men across countries (except India).

### Variation in SES inequalities between education and wealth groups

Education and wealth-related concentration indices did not always show similar patterns. For instance, both prevalence measures of angina in South Africa showed a concentration among individuals with higher wealth and among the less educated. Furthermore, there was little evidence of concentration of hypertension by education gradient in any country. This was in contrast to the gradient seen for the wealth indicator.

## Discussion

Our analysis of NCD inequalities found that self-reported diagnoses of hypertension, angina, visual impairment and depression tended to give rise to positive SES gradients, whereas symptom-based or criterion-based measures tended to display less positive gradients or even negative gradients (concentration in the lower SES groups). These differences in estimated gradients were more pronounced in China, Ghana and India (low-income and lower middle-income countries) than in Mexico, Russia and South Africa (upper-middle-income countries). Moreover, we found higher prevalence rates among women than men for most NCDs across the countries.

These findings must, however, be contextualised by important limitations. First, our data do not include younger people (below 18 years). However, this would have a limited bearing on the findings, except for asthma, as most of the conditions studied occur largely in adults. Second, we could not assess the extent of access to healthcare and other disease specific features across different SES groups as explanations for differences in self-reported diagnoses and symptom-based/criterion-based measures. Third, we incorporated standardised measures to identify NCDs to the extent possible, however, some of our symptom-based measures, namely of angina and depression, were based on self-reports of symptoms, and the reliability of such measures are also limited by lack of comprehensiveness and are subject to their own reporting biases. Another related potential source of variance in the results can arise from the variations in the performance on the standard tests between respondents of different SES groups. Furthermore, most symptoms were measured at a single time point. Individuals with angina, hypertension, visual impairment and depression may not always necessarily experience symptoms due to medication or other related reasons, resulting in incomplete case ascertainment using symptom-based measures. Finally, our symptom-based estimates did not consistently detect a higher prevalence of the NCDs than self-reported diagnoses. For example, the spirometric prevalence estimates of asthma and chronic lung diseases were lower than those of self-reported diagnoses, probably due to a misreporting of recurrent acute respiratory diseases such as asthma or COPD, and/or inability of spirometry to detect asthma that is in remission.

Our study informs methodological development for producing more robust community-based estimates of NCD prevalence in LMIC settings. We used nationally representative data in China, Ghana, India, Mexico, South Africa and Russia, and employed both self-reported diagnoses and symptom-based measures to estimate prevalence of several NCDs across SES groups. Furthermore, we used asset index and education as two distinct SES indicators.

These findings provide salient information for the ongoing debate about whether NCDs in LMICs are concentrated among the rich or the poor, the latter being the case in most high-income countries.^2^ ^33–35^ Exposure to risk factors—tobacco, high blood pressure, high blood lipids, raised blood glucose and obesity—and consequent NCDs are hypothesised to be initially greater in affluent, urban elites in LMICs.^36^ Greater exposure to these risk factors among lower SES groups^37^ may produce a reversal of SES patterning over time. However, the available evidence on SES patterning of NCDs in LMICs shows contrasting results. A study based on the World Health Survey 2004 found that NCDs are relatively more common among the lower SES groups.^6^ Some studies from India suggest that cardiometabolic diseases are prevalent among the lower SES groups,^38^ ^39^ whereas other studies reported positive associations between SES and NCDs.^33^ Evidence of positive associations of specific NCDs were reported in Ghana.^40^ In China, a higher prevalence of COPD has been reported among the less educated;^41^ negative association between educational level and obesity but a positive association between household income and central obesity was reported in rural China.^42^ Evidence from South Africa suggests that NCDs are being increasingly reported among lower SES.^43^ Furthermore, one study has reported complex intermediate patterns with an interaction between wealth and education in relation to obesity among women in LMICs, which may explain the differences in patterns by education and wealth observed in our study.^44^ Studies also found that lower SES groups had less screening and knowledge of cardiovascular risk factors, whereas those with the knowledge were more likely to make healthy behavioural changes,^45^ and educational attainment and health literacy can modify the NCDs and risk factors in LMICs.^46^ ^47^ Our findings indicated that SES gradients in NCD prevalence qualitatively differed within and between countries by type of prevalence measurement, specific NCDs and SES indicators, and thus NCDs as a category cannot be considered as diseases of affluence or of poverty.

### Suggestions for future research and policy

Our study findings require further investigation using data from a greater number of LMICs covering a wider spectrum of economic development. Longitudinal or repeat survey data would enable stronger inferences on whether ‘switching’ from a positive to negative gradient within a country occurs over time (gradient reversal). Future surveys should incorporate more comprehensive and reliable indicators of pathology such as biomarkers, and also more detailed information on the medications of each NCD so that the issue of differential healthcare across SES can be incorporated for more robust estimates of symptom-based/criterion-based measures of NCDs. Though we found that self-reported diagnoses of most NCDs across countries were generally concentrated among higher SES individuals whereas symptom-based measures were either concentrated among lower SES individuals or more evenly distributed, we did observe considerable between-country heterogeneity. The underlying reasons for this heterogeneity are a topic for further investigation. These are likely to include differential access to healthcare, public awareness of NCD symptoms, and potentially important cultural and social differences in reporting.

Our findings suggest that NCD prevalence estimates solely based on self-reported diagnoses may be misleading if used to determine the burden of disease, targeting interventions and exploring social inequalities in NCDs. Standardised diagnostic measures, using clinical assessment augmented by investigations, should be implemented in community surveys to estimate the true prevalence of NCDs and inform policies in LMICs. If this is not feasible due to resource constraints, a more comprehensive set of questions on specific NCDs should be employed to minimise possible bias due to the under-reporting and underdiagnosis among lower SES groups. Better methods of surveillance and monitoring are needed in LMICs to determine trends in incidence and mortality of specific NCDs and to evaluate health programmes for NCDs in terms of their impact on social inequalities in health.^48^

**What is already known on this subject**

- Self-reported measures of non-communicable chronic diseases (NCDs) are likely to under-estimate the true magnitude of the problem in lower socioeconomic groups.

**What this study adds**

- Self-reported diagnoses of angina, hypertension, visual impairment and depression tended to give rise to positive socioeconomic status (SES) gradients, gradients (ie concentrated among higher socioeconomic groups) whereas symptom-based or criterion-based measures of these diseases showed either less positive gradients or even concentrated among lower socioeconomic groups.
- These differences in estimated SES gradients were more pronounced in China, Ghana and India (low-middle and lower middle-income countries) than in Mexico, Russia and South Africa (upper-middle-income countries).
- Using standardised, symptom-based measures would provide more valid estimates of NCD inequalities.

## Supplementary Material

Web supplement
