# Supplementary material for: Are estimates of socioeconomic inequalities in chronic disease artefactually narrowed by self-reported measures of prevalence in low-income and middle-income countries? Findings from the WHO-SAGE survey
Source: J Epidemiol Community Health. 2014 Dec 30;69(3):218–25. doi: 10.1136/jech-2014-204621 (PMC4345525; doi:10.1136/jech-2014-204621)
Supplement: Web supplement [file jech-2014-204621-s1.pdf]

Web Appendix Table 1. Comparative descriptive statistics of China, Ghana, India, Mexico, Russia, and South Africa, 2008

	Lower and Lower Middle Income			Upper Middle and Upper Income		
	Ghana	India	China	South Africa	Mexico	Russia
GDP per capita, PPP (constant 2011 international $)	2,865	3,980	7,732	11,741	15,704	22,558
Mortality rate, infant (per 1,000 live births)	54	51	16	45	15	12
Life expectancy at birth, total (years)	60	65	75	53	76	68
Health expenditure per capita, PPP (constant 2005 international $)	84	114	282	814	892	1030
Health expenditure, public (% of total health expenditure)	58	26.3	49.9	45.8	47	66.5
Out-of-pocket health expenditure (% of total expenditure on health)	28.4	64.2	40	8.5	49.2	27.3
Physicians (per 1,000 people)	0.1	0.6	1.7	0.8	2.9	4.3
Hospital beds (per 1,000 people)	0.9	0.9	2.2	2.8	1.6	9.7
Geographic Region	Sub-Saharan Africa	South Asia	East Asia & Pacific	Sub-Saharan Africa	Latin America & Caribbean	Europe and Central Asia

Sources: World Bank http://data.worldbank.org/ ; WHO Health Statistics http://www.who.int/gho/publications/world_health_statistics/en/index.html

Web Appendix Table 2: Description of the methods of both self-reported diagnoses and symptoms-based/criterion-based measures for specific NCDs

NCDs	Self-reported diagnoses	Symptoms-based/criterion-based measures
Angina	Have you ever been diagnosed with angina or angina pectoris (a heart disease)?	Each of the following 3 conditions were to be met:<br>1) During the last 12 months, the respondents have have had experienced any pain or discomfort in the chest when walking uphill or hurry and/or ordinary pace on level ground.<br>2) The pain or discomfort had been relieved while just simply stopping the walk.<br>3) The pain or discomfort site is either 'sternum' (all level) or 'left anterior chest and left arm'.<br>To identify location of pain or discomfort, respondents were asked to choose from a picture depicting numbered panels of the upper body.
Hypertension	Have you ever been diagnosed with high blood pressure (hypertension)?	Blood pressure measurement were taken with 'Boso Medistar Wrist Blood Pressure Monitor Model S' where the systolic blood pressure, diastolic blood pressure and pulse rate were documented. Blood pressure measurements were done three times for each respondent and at least 1 minute interval has been given between each blood pressure reading. Based on the WHO criteria of hypertension for adults 18 years and older, we defined people with hypertension as those who have reported the systolic blood pressure ≥140 mmHg and/or diastolic blood pressure ≥90 mmHg. S there were three blood pressure readings, we have taken an average of the three readings, separately for systolic blood pressure and diastolic blood pressure (22, 23).
Asthma & Chronic lung diseases	Have you ever been diagnosed with asthma (an allergic respiratory disease)?<br>Have you ever been diagnosed with chronic lung disease (emphysema, bronchitis, COPD)?	Spirometry was performed after the administration of an adequate dose of short-acting inhaled bronchodilator in order to minimise variability. The respondents were asked to take a deep breath and then to blow as long and hard as he/she can into a small tube attached to the spirometer machine and then FEV1 (Forced Expiratory Volume in One Second) and FVC (Forced Vital Capacity) were documented. Three trails of spirometry test were performed. The ratio of FEV_1 (Forced Expiratory Volume in One Second) to FVC (Forced Vital Capacity) was calculated. Following the specific spirometry cut-points suggested by GOLD, those with FEV_1/FVC <70% and also had an FEV_1 < '80% predicted', in all the three trials, were termed as people with moderate and above forms of (including severe and very severe) obstructive (lung) diseases (24).
Visual impairment	In the last 5 years, were you diagnosed with a cataract in one or both of your eyes (cloudiness in the lens of the eye)?	Using the four meter distance vision Tumbling E LogMAR chart, vision tests were conducted for distance vision for both left and right eyes and the recorded the resulting 'DECIMAL' value of each eye. If the respondents use the glasses or contact lenses, the tests were conducted using them. In accordance with WHO criteria (25, 26), we applied the definition of the low vision as an approximate Log MAR equivalent of '0.5 and above' which corresponded to a 'DECIMAL' value of '0.32 and below', thus, in

		our analysis, those who score 'DECIMAL' value of '0.32 and below' in the distance vision test for at least one eye were categorized as suffering from visual impairment.
Depression	Have you ever been diagnosed with depression?	In order to categorize a person as suffering from 'moderate depression', firstly, the following two 'General criteria' (i.e., General criteria 1 and 2) should be met(27).<br>**1) General criteria 1:** At least two of the following three symptoms (conditions) must be present: i) during the last 12 months, have had a period lasting several days when felt sad, empty or depressed; ii) during the last 12 months, have had a period lasting several days when lost interest in most things usually enjoy such as personal relationships, work or hobbies/recreation, iii) during the last 12 months, have had a period lasting several days when have been feeling energy decreased or that are tired all the time.<br>**2) General criteria 2**: The depressive episode should last for at least 2 weeks.<br>Secondly, if the above stated conditions are met, then a total of at least six conditions (symptoms) should be present to term a person as having 'moderate depression'. These six conditions can be from the above stated three conditions from 'General criteria 1', and/or, from the following 'Seven Sub-conditions'<br>**3) Seven Sub-conditions**<br>i) During this period, person did feel negative about him/herself or like he/she had lost confidence and/or did frequently feel hopeless - that there was no way to improve things,<br>ii) During this period, person did feel anxious and worried most days,<br>iii) During this period, the person did think of death, or wish he/she were dead, and/or did he/she ever try to end own life,<br>iv) During this period, did he/she have any difficulties concentrating; for example, listening to others, working, watching TV, listening to the radio, and/or did notice any slowing down in his/her thinking,<br>v) He/she did notice any slowing down in his/her moving around, and/or he/she were so restless or jittery nearly every day that he/she paced up and down and couldn't sit still,<br>vi) He/she did notice any problems falling asleep, and/or, he/she did notice any problems waking up too early, and<br>vii) During this period, he/she did lose his/her appetite.

Source: author's compilation from various sources

Web Appendix Table 3: Age-adjusted prevalence rate (in %, with 95% CI) of NCDs across wealth quintiles, measured through self-reported diagnoses and symptoms-based/criterion-based measure, among adult population in China, Ghana, India, Mexico, Russia, and South Africa

	**Angina**		**Hypertension**		**Asthma and chronic lung diseases**		**Visual impairment**		**Depression**	
	Symptoms-based measure	Self-reported diagnoses	Criterion-based measure	Self-reported diagnoses	Criterion-based measure	Self-reported diagnoses	Criterion-based measure	Self-reported diagnoses	Symptoms-based measure	Self-reported diagnoses
**Ghana**										
Lowest quintile	4.4[2.5;6.3]	1.7[0.2;3.2]	34.8[29.7;39.9]	2.5[1.3;3.7]	0.9[0.0;1.8]	1.9[0.3;3.6]	13.2[10.3;16.1]	1.4[0.4;2.4]	4.3[2.1;6.5]	0.2[0.1;0.4]
2nd	4.9[3.1;6.7]	0.8[0.4;1.1]	41.9[36.3;47.6]	4.0[2.8;5.2]	2.8[0.9;4.8]	4.7[2.1;7.2]	18.7[14.8;22.6]	1.6[0.8;2.3]	5.2[3.2;7.3]	0.6[0.3;0.8]
3rd	4.7[2.9;6.4]	1.2[0.4;2.1]	47.1[41.8;52.3]	8.5[5.3;11.6]	0.9[0.0;1.8]	1.3[0.8;1.9]	11.4[8.6;14.2]	1.4[0.6;2.1]	3.9[1.8;5.9]	1.0[0.2;1.7]
4th	3.0[1.1;4.9]	2.9[1.1;4.6]	46.3[40.8;51.7]	10.0[7.2;12.9]	2.2[0.4;3.9]	2.2[0.7;3.6]	10.4[7.1;13.8]	2.7[1.7;3.6]	2.8[1.2;4.4]	0.6[0.2;0.9]
Highest quintile	1.7[0.6;2.9]	1.1[0.6;1.5]	40.8[35.0;46.5]	13.9[10.4;17.4]	1.0[0.5;1.5]	1.6[0.8;2.4]	15.5[11.6;19.3]	3.9[2.4;5.3]	6.1[3.1;9.1]	1.1[-0.2;2.3]
**India**										
Lowest quintile	10.7[8.7;12.7]	3.0[2.0;4.0]	18.4[15.8;21.0]	5.3[3.9;6.6]	5.3[3.9;6.7]	7.5[5.7;9.3]	19.5[17.2;21.7]	5.1[4.1;6.1]	10.1[8.3;12.0]	2.4[1.4;3.3]
2nd	7.6[5.9;9.2]	2.4[1.5;3.3]	17.5[15.2;19.9]	6.7[5.4;8.1]	5.7[4.4;7.0]	5.0[3.7;6.3]	17.6[15.6;19.7]	5.9[4.8;7.1]	9.5[7.9;11.2]	3.0[1.8;4.2]
3rd	7.0[5.5;8.5]	3.9[2.6;5.2]	18.8[16.5;21.2]	9.8[7.9;11.7]	4.9[3.5;6.2]	5.9[4.3;7.4]	17.2[15.3;19.2]	6.0[4.9;7.2]	10.3[8.5;12.0]	4.3[2.9;5.7]
4th	6.3[4.7;7.9]	3.0[2.0;4.0]	19.7[17.2;22.1]	9.7[8.1;11.3]	5.0[3.7;6.3]	5.2[3.7;6.7]	17.3[15.3;19.3]	6.5[5.2;7.7]	6.8[5.3;8.2]	4.8[3.4;6.2]
Highest quintile	3.4[2.4;4.4]	2.7[1.9;3.4]	21.3[18.5;24.2]	15.2[13.0;17.4]	5.0[3.7;6.3]	5.6[3.7;7.5]	12.4[10.9;13.9]	6.6[5.5;7.7]	5.6[4.1;7.0]	2.9[1.8;3.9]
**China**										
Lowest quintile	1.7[0.9;2.5]	3.0[2.1;3.9]	38.6[35.1;42.2]	9.6[7.7;11.4]	1.5[1.0;1.9]	4.1[3.0;5.2]	16.0[13.3;18.6]	2.9[1.5;4.2]	1.2[0.6;1.8]	0.1[0.0;0.1]
2nd	1.4[0.7;2.2]	3.9[2.9;5.0]	37.9[34.0;41.8]	9.0[7.3;10.8]	1.1[0.8;1.5]	4.1[2.9;5.3]	12.6[10.2;15.0]	2.3[1.8;2.7]	1.7[0.3;3.0]	0.1[0.0;0.1]
3rd	1.9[0.6;3.3]	3.0[2.2;3.9]	40.5[35.8;45.1]	12.6[9.9;15.4]	1.1[0.6;1.5]	5.3[3.1;7.5]	14.4[11.5;17.3]	2.6[2.1;3.1]	0.4[-0.1;1.0]	1.1[-0.2;2.4]
4th	0.8[0.4;1.2]	4.3[2.6;6.1]	35.8[31.3;40.2]	14.1[11.1;17.1]	1.3[0.3;2.2]	3.6[2.5;4.8]	14.0[11.2;16.8]	2.7[2.3;3.2]	1.2[0.1;2.3]	0.2[0.0;0.4]
Highest quintile	1.0[0.3;1.8]	4.3[2.4;6.1]	34.6[29.9;39.3]	13.6[11.0;16.2]	1.4[0.6;2.1]	3.3[2.2; 4.4]	15.5[12.1;18.9]	3.9[2.6;5.2]	0.4[-0.2;0.9]	0.4[-0.2;0.9]

**South Africa**										
Lowest quintile	1.0[0.4;1.6]	1.0[0.4;1.6]	45.0[38.7;51.2]	5.9[4.9;6.9]	0.7[0.4;1.0]	4.2[0.9;7.5]	20.0[15.4;24.6]	3.2[0.8;5.6]	6.2[2.3;10.2]	0.6[0.1;1.2]
2nd	1.5[0.8;2.2]	1.1[0.6;1.5]	57.2[51.0;63.4]	8.7[6.7;10.7]	1.2[0.5;1.9]	2.3[1.3;3.4]	20.1[15.4;24.7]	0.8[0.5;1.2]	4.3[0.0;8.6]	4.4[0.1;8.6]
3rd	1.7[0.5;2.8]	3.2[1.3;5.1]	59.4[54.8;64.0]	15.2[10.8;19.7]	6.2[2.4;10.1]	2.8[1.4;4.3]	13.7[11.8;15.7]	3.8[2.7;5.0]	3.9[1.0;6.7]	5.8[3.1;8.4]
4th	5.1[4.0;6.1]	3.1[2.1;4.2]	51.0[45.6;56.3]	13.4[10.8;16.0]	0.5[0.1;10.9]	5.2[3.1;7.3]	18.9[15.0;22.8]	1.2[0.7;1.6]	5.6[3.6;7.5]	5.5[3.4;7.6]
Highest quintile	0.5[0.1;1.0]	10.7[8.7;12.8]	53.0[47.6;58.4]	12.0[9.2;14.8]	0.4[0.0;0.8]	2.7[0.8;4.6]	25.7[20.9;30.6]	3.5[1.4;5.5]	1.2[0.8;1.6]	5.4[2.5;8.4]
**Mexico**										
Lowest quintile	10.8[7.8;13.9]	0.7[0.5;0.9]	25.4[21.8;29.1]	11.9[9.2;14.6]	0.2[0.0;0.4]	5.0[3.6;6.3]	30.5[23.9;37.1]	12.7[7.0;18.3]	7.5[5.8;9.2]	12.2[9.6;14.8]
2nd	8.3[6.0;10.5]	0.6[0.3;0.9]	28.2[22.1;34.3]	8.5[6.9;10.2]	0.3[-0.4;1.1]	10.9[7.5;14.3]	17.7[14.4;21.1]	2.9[2.2;3.7]	1.8[0.6;3.0]	7.5[4.7;10.2]
3rd	6.5[3.3;9.8]	0.6[0.2;1.0]	32.6[27.5;37.6]	11.6[9.1;14.1]	0.3[0.1;0.5]	2.7[1.3;4.2]	22.0[18.3;25.8]	4.3[2.9;5.7]	10.1[6.9;13.4]	13.5[9.5;17.4]
4th	7.4[5.5;9.4]	1.7[-0.1;3.5]	31.2[25.0;37.4]	22.1[17.6;26.6]	0.1[-0.1;0.4]	5.5[1.6;9.3]	27.8[22.0;33.6]	3.4[2.4;4.4]	8.2[3.8;12.6]	12.3[8.0;16.6]
Highest quintile	4.0[1.5;6.5]	1.2[0.1;2.2]	29.8[23.0;36.5]	14.1[10.3;17.8]	4.0[-0.7;8.6]	3.9[1.0;6.9]	17.1[14.2;20.0]	3.7[1.6;5.8]	6.5[3.3;9.6]	8.3[4.6;11.9]
**Russia**										
Lowest quintile	13.2[10.7;15.7]	18.4[15.3;21.4]	45.5[41.7;49.4]	33.8[29.9;37.8]	0.5[0.2;0.8]	11.2[8.9;13.5]	19.4[16.4;22.3]	10.6[8.1;13.0]	3.7[2.6;4.9]	3.1[1.7;4.5]
2nd	18.9[15.6;22.1]	27.8[24.2;31.4]	41.6[38.1;45.1]	39.3[35.7;42.9]	0.2[0.0;0.5]	17.8[14.5;21.1]	26.8[23.3;30.2]	9.7[7.6;11.7]	4.8[2.4;7.2]	2.3[1.3;3.3]
3rd	18.4[14.5;22.2]	28.4[24.1;32.7]	46.5[42.2;50.8]	41.3[36.5;46.1]	0.1[0.0;0.1]	17.0[13.8;20.2]	26.5[22.7;30.4]	10.2[7.6;12.7]	4.3[2.0;6.6]	5.0[2.6;7.4]
4th	13.9[10.8;17.1]	23.2[19.2;27.3]	43.6[39.5;47.8]	35.5[31.7;39.3]	0.9[0.1;1.7]	15.6[12.1;19.2]	21.8[17.9;25.8]	9.7[7.3;12.0]	5.8[3.6;8.1]	3.7[1.6;5.7]
Highest quintile	13.8[10.5;17.0]	21.3[17.1;25.4]	35.2[30.4;40.0]	39.7[35.3;44.1]	1.0[-0.1;2.0]	17.5[13.9;21.0]	20.1[17.2;23.0]	8.8[6.6;11.0]	2.6[1.0;4.2]	3.8[1.6;6.0]
All Sample	15.7[13.5;17.9]	23.8[21.2;26.3]	41.9[39.1;44.6]	36.7[33.9;39.4]	0.3[0.1;0.4]	15.0[12.8;17.2]	24.3[21.9;26.8]	9.6[8.1;11.2]	4.8[3.3;6.3]	3.9[2.4;5.3]

Source: WHO SAGE survey, 2007-10

Web Appendix Table 4: Age-adjusted prevalence rate (in %, with 95% CI) of NCDs across educational level, measured through self-reported diagnoses and symptoms-based/criterion-based measures, among adult population in China, Ghana, India, Mexico, Russia, and South Africa

	Angina		Hypertension		Asthma and chronic lung diseases		Visual impairment		Depression	
	Symptoms-based measure	Self-reported diagnoses	Criterion-based measure	Self-reported diagnoses	Criterion-based measure	Self-reported diagnoses	Criterion-based measure	Self-reported diagnoses	Symptoms-based measure	Self-reported diagnoses
**Ghana**										
No Formal Schooling	6.2[4.0;8.4]	2.0[0.6;3.5]	42.2[37.0;47.3]	4.7[2.5;6.9]	1.7[0.6;2.8]	2.5[1.9;3.1]	17.2[13.8;20.7]	1.4[0.9;2.0]	4.1[2.4;5.9]	0.3[0.2;0.5]
Less than primary school	4.1[2.4;5.9]	0.9[0.2;1.5]	39.8[33.9;45.7]	6.2[3.4;9.1]	3.4[0.5;6.3]	3.8[2.0;5.7]	14.6[10.6;18.6]	1.8[1.1;2.4]	3.4[1.8;5.0]	1.0[0.5;1.5]
Primary school completed	3.9[2.1;5.7]	0.9[0.5;1.4]	47.9[42.2;53.5]	10.1[6.4;13.8]	1.5[-0.2;3.2]	2.8[0.7;5.0]	13.6[10.2;17.0]	1.8[1.3; 2.4]	3.6[1.5;5.7]	0.9[0.2;1.7]
Secondary high school completed	1.4[0.7;2.2]	1.7[0.7;2.7]	42.9[38.4;47.4]	9.7[7.4;12.0]	1.5[0.5;2.6]	2.8[1.6;4.0]	12.1[9.4;14.8]	3.8[2.3;5.3]	4.1[2.4;5.7]	0.2[0.0;0.3]
College completed or above	1.4[0.6;2.2]	1.1[0.2;2.0]	35.8[31.4;40.2]	14.3[10.3;18.2]	0.8[0.1;1.6]	1.3[0.7;1.9]	11.6[10.3;13.0]	3.0[1.1;5.0]	4.0[1.2;6.7]	5.3[4.9;5.7]
**India**										
No Formal Schooling	8.5[7.2;9.8]	2.7[1.9;3.4]	19.3[17.2;21.3]	8.6[7.4;9.8]	6.3[5.0;7.5]	5.1[4.2;6.0]	18.0[16.7;19.3]	5.7[5.0;6.4]	10.5[9.1;11.9]	2.6[1.9;3.3]
Less than primary school	8.1[6.0;10.1]	3.6[2.3;4.9]	18.7[15.3;22.0]	7.9[6.0;9.7]	2.8[1.9;3.7]	6.6[4.5;8.7]	17.3[14.5;20.1]	6.9[5.4;8.4]	8.3[6.2;10.3]	3.7[2.1;5.2]
Primary school completed	6.5[4.8;8.1]	2.9[2.0;3.8]	21.6[18.8;24.4]	8.0[6.5;9.4]	4.9[3.6; 6.2]	5.3[3.8;6.8]	17.9[15.5;20.4]	4.8[3.9;5.7]	7.1[5.4;8.9]	3.0[1.9;4.1]
Secondary high school completed	5.1[3.7;6.4]	3.8[2.7;4.9]	17.7[15.5;19.9]	12.0[10.1;13.9]	5.3[4.0;6.6]	6.5[4.8;8.1]	14.0[12.3;15.7]	7.3[6.0;8.7]	6.4[5.0;7.8]	3.3[2.3;4.3]
College completed or above	2.3[0.6;4.1]	2.9[1.3;4.5]	19.8[16.0;23.5]	14.4[11.5;17.3]	3.7[2.2;5.3]	4.7[2.4;7.1]	10.9[8.4;13.3]	5.0[3.4;6.6]	4.8[1.8;7.8]	3.9[2.1;5.7]
**China**										
No Formal Schooling	1.1[0.8;1.3]	2.2[1.8;2.5]	51.3[45.3;57.4]	14.4[10.7;18.2]	1.3[1.0;1.5]	2.7[2.3;3.0]	17.1[13.2;21.1]	2.2[1.9;2.5]	0.8[0.2;1.4]	0.1[0.0;0.2]
Less than primary school	0.5[0.3;0.7]	1.8[1.2;2.4]	34.9[29.9;40.0]	12.4[8.7;16.1]	1.5[0.5;2.5]	5.1[2.3;7.9]	16.3[12.6;20.0]	3.0[1.7;4.3]	0.7[-0.2;1.6]	0.7[-0.4;1.9]
Primary school completed	1.3[0.3;2.2]	4.4[2.4;6.5]	42.1[37.4;46.9]	10.9[8.1;13.7]	0.8[0.6;1.1]	6.8[4.1;9.5]	11.4[9.1;13.8]	2.9[1.3;4.5]	0.4[0.1;0.8]	0.3[-0.2;0.8]
Secondary high school completed	1.6[1.0; 2.2]	4.5[3.6;5.4]	36.7[34.2;39.3]	12.3[11.0;13.7]	1.1[0.6;1.6]	3.5[2.8;4.2]	13.4[11.6;15.2]	3.0[2.5;3.5]	1.3[0.6;1.9]	0.3[0.0;0.7]
College completed or above	0.8[0.4;1.2]	3.4[2.2;4.6]	32.3[27.0;37.6]	12.4[9.2;15.6]	1.4[0.2;2.6]	6.9[3.5;10.3]	14.9[10.6;19.2]	3.3[2.6;4.0]	0.6[-0.3;1.6]	0.1[-0.1;0.2]

**South Africa**										
No Formal Schooling	2.6[1.7;3.6]	1.6[0.9;2.2]	65.4[57.3;73.5]	12.5[10.9;14.1]	1.4[0.0;2.7]	2.6[1.7;3.5]	17.6[12.7;22.5]	4.7[1.6;7.8]	3.7[0.0;7.4]	0.7[0.3;1.2]
Less than primary school	2.6[0.6;4.6]	2.6[0.6;4.6]	57.0[50.6;63.5]	17.6[12.3;23.0]	3.7[-1.0;8.3]	6.9[1.9;11.9]	16.9[13.2;20.7]	1.1[0.6;1.5]	3.2[0.3;6.0]	1.8[-0.2;3.7]
Primary school completed	4.5[4.0;4.9]	7.7[5.8;9.7]	54.6[49.4;59.8]	12.0[8.9;15.1]	1.2[0.5;1.9]	6.1[3.2;8.9]	17.8[14.6;20.9]	1.7[1.2;2.3]	4.3[1.6;6.9]	3.6[1.2;6.0]
Secondary high school completed	0.7[0.2;1.2]	2.9[1.4;4.4]	47.8[41.5;54.1]	10.8[8.4;13.2]	0.3[0.0;0.6]	3.2[1.6;4.9]	22.6[17.6;27.6]	3.8[1.4;6.2]	4.1[0.7;7.6]	6.0[2.1;9.9]
College completed or above	1.2[-0.2;2.6]	4.5[3.1;5.8]	60.5[57.6;63.3]	10.2[6.3;14.2]	0.2[-0.1;0.4]	0.6[-0.1;1.3]	23.5[20.8;26.1]	1.8[1.1;2.5]	0.3[0.0;0.6]	9.0[7.8;10.3]
**Mexico**										
No Formal Schooling	ǂ	ǂ	ǂ	ǂ	ǂ	ǂ	ǂ	ǂ	ǂ	ǂ
Less than primary school	6.6[3.9;9.4]	1.9[0.0;3.9]	24.7[21.6;27.8]	13.0[10.4;15.7]	0.2[0.0;0.3]	2.0[0.9;3.0]	25.8[22.9;28.7]	4.3[2.9;5.6]	3.2[1.9;4.5]	7.5[5.7;9.4]
Primary school completed	7.9[4.9;10.8]	4.2[2.1;6.3]	29.5[24.1;34.9]	16.5[12.9;20.0]	1.5[-1.3;4.2]	9.2[4.8;13.7]	24.9[20.6;29.1]	3.3[2.3;4.4]	4.6[2.2;7.0]	12.0[8.4;15.7]
Secondary high school completed	6.3[3.2;9.3]	1.0[0.6;1.5]	28.7[23.4;34.0]	11.7[9.0;14.4]	0.9[-0.8;2.5]	3.5[1.6;5.4]	25.2[20.4;30.0]	2.8[1.5;4.0]	7.2[4.1;10.4]	10.0[6.8;13.3]
College completed or above	2.1[0.3;3.9]	0.2[0.0;0.4]	26.6[22.8;30.5]	15.8[12.5;19.1]	0.6[0.1;1.1]	3.9[2.0;5.8]	16.2[13.8;18.6]	8.8[7.2;10.5]	9.6[6.4;12.9]	12.8[8.9;16.7]
**Russia**										
No Formal Schooling	32.4[28.2;36.5]	29.3[25.1;33.5]	67.6[63.5;71.8]	79.2[75.8; 82.6]	0.0[0.0;0.0]	16.1[12.5;19.6]	43.2[39.8;46.7]	24.3[20.4;28.3]	12.3[9.7;14.9]	0.0[0.0;0.0]
Less than primary school	30.6[24.4;36.7]	31.5[24.9;38.2]	68.4[62.4;74.5]	73.6[68.8;78.4]	3.8[0.0;0.0]	29.1[22.7;35.5]	40.4[35.7;45.0]	16.5[12.4;20.7]	13.9[11.3;16.4]	2.7[0.0;5.4]
Primary school completed	32.0[25.8;38.2]	35.0[30.0; 40.1]	64.9[58.9;71.0]	53.3[47.1;59.6]	2.6[0.8;4.3]	26.9[21.3;32.5]	37.0[31.4;42.7]	12.3[8.8;15.9]	3.1[0.8;5.4]	1.0[0.0;1.9]
Secondary high school completed	15.8[13.1;18.4]	24.0[21.2;26.9]	44.5[41.4;47.7]	39.5[36.4; 42.6]	0.5[0.2;0.8]	16.4[13.8;19.1]	25.1[22.3;27.8]	9.4[7.5;11.3]	4.8[3.1;6.5]	4.3[2.5;6.1]
College completed or above	13.5[10.7;16.3]	22.8[20.2;25.5]	33.3[29.9;36.7]	31.5[28.2;34.9]	0.0[0.0;0.1]	9.7[7.4;12.0]	17.3[13.9;20.6]	13.2[10.3;16.1]	3.8[1.8;5.9]	4.7[2.6; 6.8]

Notes:Source: WHO SAGE survey, 2007-10. ǂ *no observations were reported in the survey corresponding to 'No Formal schooling' in the sample in Mexico.*

Web Appendix Table 5: Unadjusted and Adjusted Odds Ratio (with 95% CI) of self-reported diagnoses and symptoms-based/criterion-based measures of NCDs among adult population in China, Ghana, and India.

	**Ghana**						**India**						**China**					
**Angina**																		
	**Symptom-based measure**			**Self-reported diagnoses**			**Symptom-based measure**			**Self-reported diagnoses**			**Symptom-based measure**			**Self-reported diagnoses**		
	**Model 1**	**Model 2**	**Model 3**	**Model 1**	**Model 2**	**Model 3**	**Model 1**	**Model 2**	**Model 3**	**Model 1**	**Model 2**	**Model 3**	**Model 1**	**Model 2**	**Model 3**	**Model 1**	Model 2	Model 3
Male	0.54* [0.32,0.89]	0.54* [0.32,0.90]	0.73 [0.31,1.71]	0.39** [0.20,0.77]	0.31*** [0.16,0.61]	0.20* [0.055,0.70]	0.78 [0.60,1.00]	0.72* [0.55,0.95]	0.95 [0.63,1.42]	1.12 [0.79,1.59]	1.08 [0.76,1.54]	1.40 [0.86,2.28]	0.57 [0.30,1.07]	0.74 [0.37,1.46]	0.75 [0.34,1.64]	0.69 [0.45,1.06]	0.73 [0.46,1.14]	0.66 [0.37,1.18]
Asset	0.38*** [0.23,0.62]	0.51* [0.27,0.96]		1.01 [0.40,2.55]	2.01 [0.60,6.71]		0.47*** [0.37,0.60]	0.45*** [0.35,0.58]		1.10 [0.81,1.49]	0.99 [0.73,1.34]		0.39** [0.21,0.72]	0.47* [0.23,0.98]		0.87 [0.55,1.38]	1.21 [0.76,1.93]	
Education	0.89 [0.79,1.00]		0.91 [0.82,1.02]	1.08 [0.99,1.18]		1.16** [1.04,1.29]	0.92** [0.86,0.98]		0.94* [0.89,1.00]	0.98 [0.94,1.04]		1.00 [0.95,1.05]	0.97 [0.90,1.04]		0.99 [0.92,1.07]	0.98 [0.93,1.03]		1.04 [0.95,1.13]
**Hypertension**																		
	**Criterion-based measure**			**Self-reported diagnoses**			**Criterion-based measure**			**Self-reported diagnoses**			**Criterion-based measure**			**Self-reported diagnoses**		
	**Model 1**	**Model 2**	**Model 3**	**Model 1**	**Model 2**	**Model 3**	**Model 1**	**Model 2**	**Model 3**	**Model 1**	**Model 2**	**Model 3**	**Model 1**	**Model 2**	**Model 3**	**Model 1**	**Model 2**	**Model 3**
Male	1.05 [0.83,1.34]	1.03 [0.80,1.32]	1.35 [0.96,1.90]	0.65 [0.42,1.01]	0.54** [0.34,0.86]	0.52 [0.26,1.03]	1.05 [0.90,1.23]	1.03 [0.88,1.21]	1.07 [0.85,1.36]	0.61*** [0.49,0.75]	0.56*** [0.44,0.70]	0.45*** [0.34,0.61]	1.29** [1.08,1.54]	1.31** [1.08,1.59]	1.34** [1.09,1.66]	0.83 [0.66,1.05]	0.89 [0.69,1.14]	0.85 [0.63,1.13]
Asset	1.34* [1.03,1.74]	1.17 [0.87,1.59]		3.82*** [2.38,6.15]	3.53*** [2.08,6.00]		1.16 [0.98,1.37]	1.05 [0.87,1.25]		2.27*** [1.83,2.81]	2.13*** [1.71,2.65]		0.70*** [0.57,0.86]	1.06 [0.84,1.33]		0.93 [0.73,1.17]	1.52** [1.14,2.01]	
Education	0.96 [0.93,1.00]		0.95* [0.91,0.99]	1.06 [0.97,1.15]		1.05 [0.96,1.16]	0.99 [0.96,1.02]		1.00 [0.97,1.03]	1.03 [1.00,1.07]		1.09*** [1.05,1.12]	0.92*** [0.90,0.95]		1.00 [0.96,1.03]	0.95** [0.92,0.98]		1.01 [0.96,1.05]
**Asthma and chronic lung disease**																		
	**Criterion-based measure**			**Self-reported diagnoses**			**Criterion-based measure**			**Self-reported diagnoses**			**Criterion-based measure**			**Self-reported diagnoses**		
	**Model 1**	**Model 2**	**Model 3**	**Model 1**	**Model 2**	**Model 3**	**Model 1**	**Model 2**	**Model 3**	**Model 1**	**Model 2**	**Model 3**	**Model 1**	**Model 2**	**Model 3**	**Model 1**	**Model 2**	**Model 3**
Male	0.80 [0.32,1.99]	0.79 [0.32,1.96]	0.66 [0.16,2.70]	1.08 [0.52,2.27]	1.09 [0.53,2.25]	0.89 [0.39,2.04]	0.87 [0.66,1.14]	0.81 [0.62,1.07]	0.68* [0.47,0.98]	1.51* [1.09,2.09]	1.43* [1.03,1.99]	1.26 [0.75,2.12]	0.42*** [0.25,0.69]	0.39** [0.22,0.69]	0.41** [0.22,0.80]	1.84 [0.99,3.44]	2.06* [1.07,3.94]	2.43* [1.02,5.81]
Asset	1.04 [0.54,2.03]	0.92 [0.54,1.56]		0.99 [0.59,1.64]	0.82 [0.45,1.48]		0.88 [0.66,1.16]	0.91 [0.67,1.24]		1.11 [0.74,1.65]	1.03 [0.68,1.56]		0.65 [0.28,1.49]	0.99 [0.54,1.80]		0.89 [0.37,2.15]	1.00 [0.47,2.13]	
Education	0.98 [0.86,1.12]		0.98 [0.86,1.13]	0.95 [0.88,1.03]		0.96 [0.89,1.04]	0.97 [0.93,1.01]		1.00 [0.96,1.04]	1.01 [0.94,1.09]		1.03 [0.97,1.11]	0.95 [0.87,1.04]		0.97 [0.91,1.04]	1.00 [0.88,1.12]		0.98 [0.83,1.17]
**Visual impairment**																		

	Criterion-based measure			Self-reported diagnoses			Criterion-based measure			Self-reported diagnoses			Criterion-based measure			Self-reported diagnoses		
	**Model 1**	**Model 2**	**Model 3**	**Model 1**	**Model 2**	**Model 3**	**Model 1**	**Model 2**	**Model 3**	**Model 1**	**Model 2**	**Model 3**	**Model 1**	**Model 2**	**Model 3**	**Model 1**	**Model 2**	**Model 3**
Male	1.15<br>[0.84,1.58]	1.10<br>[0.79,1.53]	1.39<br>[0.79,2.45]	0.58<br>[0.32,1.08]	0.49*<br>[0.26,0.94]	0.28*<br>[0.093,0.82]	0.75***<br>[0.65,0.87]	0.62***<br>[0.52,0.74]	0.72*<br>[0.54,0.95]	0.94<br>[0.77,1.15]	0.85<br>[0.68,1.06]	0.86<br>[0.61,1.23]	0.73**<br>[0.58,0.92]	0.74*<br>[0.58,0.94]	0.80<br>[0.61,1.05]	0.66<br>[0.43,1.01]	0.73<br>[0.46,1.17]	0.82<br>[0.44,1.56]
Asset	0.78<br>[0.55,1.09]	0.93<br>[0.63,1.38]		2.37**<br>[1.28,4.42]	2.63***<br>[1.48,4.68]		0.81**<br>[0.70,0.94]	0.72***<br>[0.60,0.86]		1.37**<br>[1.12,1.67]	1.28*<br>[1.04,1.58]		0.66**<br>[0.50,0.87]	0.84<br>[0.64,1.11]		0.68<br>[0.40,1.14]	1.14<br>[0.69,1.88]	
Education	0.98<br>[0.91,1.05]		0.98<br>[0.91,1.06]	1.23**<br>[1.09,1.39]		1.25***<br>[1.10,1.43]	0.92***<br>[0.89,0.95]		0.97*<br>[0.93,1.00]	0.96*<br>[0.92,1.00]		1.02<br>[0.97,1.06]	1.01<br>[0.96,1.05]		1.03<br>[0.99,1.08]	0.93*<br>[0.88,0.99]		0.98<br>[0.89,1.08]
**Depression**																		
	**Symptom-based measure**			**Self-reported diagnoses**			**Symptom-based measure**			**Self-reported diagnoses**			**Symptom-based measure**			**Self-reported diagnoses**		
	**Model 1**	**Model 2**	**Model 3**	**Model 1**	**Model 2**	**Model 3**	**Model 1**	**Model 2**	**Model 3**	**Model 1**	**Model 2**	**Model 3**	**Model 1**	**Model 2**	**Model 3**	**Model 1**	**Model 2**	**Model 3**
Male	0.89<br>[0.52,1.53]	0.81<br>[0.46,1.41]	1.42<br>[0.56,3.61]	1.50<br>[0.54,4.18]	1.30<br>[0.47,3.62]	2.56<br>[0.62,10.6]	0.79*<br>[0.63,0.99]	0.74*<br>[0.59,0.94]	0.97<br>[0.69,1.38]	1.78***<br>[1.26,2.51]	1.71**<br>[1.22,2.39]	1.44<br>[0.89,2.33]	1.18<br>[0.54,2.61]	1.23<br>[0.56,2.70]	1.51<br>[0.63,3.62]	0.75<br>[0.15,3.84]	0.81<br>[0.18,3.78]	0.89<br>[0.22,3.58]
Asset	1.25<br>[0.65,2.43]	1.01<br>[0.53,1.91]		2.08<br>[0.51,8.55]	2.34<br>[0.91,6.00]		0.59***<br>[0.48,0.73]	0.56***<br>[0.45,0.71]		1.15<br>[0.82,1.61]	1.17<br>[0.81,1.68]		0.59<br>[0.24,1.47]	0.48<br>[0.22,1.01]		3.10<br>[0.72,13.3]	2.68<br>[0.35,20.6]	
Education	0.93<br>[0.83,1.04]		0.91<br>[0.81,1.04]	0.95<br>[0.86,1.05]		0.93*<br>[0.87,0.99]	0.93**<br>[0.89,0.98]		0.96<br>[0.91,1.00]	0.99<br>[0.93,1.05]		1.00<br>[0.94,1.06]	0.98<br>[0.89,1.08]		0.98<br>[0.87,1.11]	0.90<br>[0.74,1.09]		0.83<br>[0.60,1.13]

Notes: i)95% confidence intervals in brackets; ii) * $p < 0.05$, ** $p < 0.01$, *** $p < 0.001$; iii) dependent variable is symptom-based/criterion-based measure and self-reported diagnoses of each NCD, and is defined as 1 if the respondent had the NCD, and 0 if otherwise; iv) Male is dichotomous variable with female as reference category, asset is defined as the asset index of the household, and education is defined as years of education; v) Model 1 estimated unadjusted odds ratio by regressing each independent variable on NCD prevalence separately; Model 2 is adjusted for rural-urban, age, gender and asset, where Model 3 is adjusted for rural-urban, age, gender and education. Model 2 and 3 of symptom-based/criterion-based measures of each was also adjusted with a dichotomous variable of whether the respondent has taken medication/treatment or not.

Web Appendix Table 6: Unadjusted and Adjusted Odds Ratio (with 95% CI) of self-reported diagnoses and symptoms-based/criterion-based measures of NCDs among adult population in South Africa, Mexico, and Russia.

	**South Africa**						**Mexico**						**Russia**					
**Angina**																		
	**Symptom-based measure**			**Self-reported diagnoses**			**Symptom-based measure**			**Self-reported diagnoses**			**Symptom-based measure**			**Self-reported diagnoses**		
	**Model 1**	**Model 2**	**Model 3**	**Model 1**	**Model 2**	**Model 3**	**Model 1**	**Model 2**	**Model 3**	**Model 1**	**Model 2**	**Model 3**	**Model 1**	**Model 2**	**Model 3**	**Model 1**	**Model 2**	**Model 3**
Male	0.24*** [0.11,0.52]	0.29** [0.12,0.69]	0.45 [0.18,1.13]	0.28** [0.13,0.62]	0.28** [0.13,0.61]	0.20*** [0.07,0.52]	0.44* [0.20,0.99]	0.49 [0.21,1.14]	0.43 [0.18,1.01]	0.29* [0.10,0.83]	0.28* [0.10,0.79]	0.31* [0.10,0.96]	0.81 [0.52,1.27]	1.05 [0.61,1.79]	1.22 [0.72,2.08]	0.84 [0.59,1.20]	0.94 [0.63,1.41]	0.94 [0.62,1.42]
Asset	0.97 [0.60,1.57]	1.30 [0.62,2.71]		5.33** [1.58,18.0]	6.23** [1.85,21.0]		0.36* [0.15,0.87]	0.30* [0.11,0.80]		1.85 [0.42,8.11]	2.29 [0.51,10.3]		0.60* [0.40,0.90]	0.94 [0.52,1.70]		0.69* [0.47,1.00]	1.17 [0.75,1.82]	
Education	0.86 [0.72,1.03]		0.93 [0.76,1.13]	0.93 [0.82,1.06]		0.96 [0.84,1.11]	0.88 [0.77,1.01]		0.88 [0.77,1.01]	0.87* [0.78,0.97]		0.96 [0.88,1.05]	0.86*** [0.81,0.91]		0.96 [0.89,1.04]	0.90*** [0.85,0.95]		1.02 [0.96,1.08]
**Hypertension**																		
	**Criterion-based measure**			**Self-reported diagnoses**			**Criterion-based measure**			**Self-reported diagnoses**			**Criterion-based measure**			**Self-reported diagnoses**		
	**Model 1**	**Model 2**	**Model 3**	**Model 1**	**Model 2**	**Model 3**	**Model 1**	**Model 2**	**Model 3**	**Model 1**	**Model 2**	**Model 3**	**Model 1**	**Model 2**	**Model 3**	**Model 1**	**Model 2**	**Model 3**
Male	1.33 [0.77,2.32]	1.44 [0.82,2.53]	2.11* [1.02,4.38]	0.49** [0.32,0.76]	0.45*** [0.29,0.68]	0.35*** [0.20,0.58]	1.12 [0.65,1.92]	1.37 [0.79,2.40]	1.35 [0.75,2.43]	0.54* [0.31,0.93]	0.46** [0.27,0.76]	0.45** [0.26,0.79]	0.96 [0.71,1.30]	1.36 [0.95,1.93]	1.22 [0.86,1.73]	0.61** [0.44,0.83]	0.62* [0.43,0.90]	0.65* [0.45,0.94]
Asset	0.88 [0.54,1.43]	0.99 [0.63,1.56]		1.57* [1.03,2.37]	1.78** [1.22,2.60]		0.88 [0.47,1.64]	0.80 [0.39,1.62]		2.04* [1.09,3.80]	2.75** [1.50,5.04]		0.46*** [0.31,0.67]	0.50** [0.32,0.78]		0.74 [0.51,1.07]	1.25 [0.82,1.88]	
Education	0.93 [0.86,1.00]		0.97 [0.90,1.05]	0.90** [0.84,0.97]		0.98 [0.92,1.05]	0.97 [0.92,1.03]		1.04 [0.98,1.11]	0.95 [0.86,1.05]		1.03 [0.93,1.14]	0.88*** [0.84,0.92]		0.97 [0.92,1.02]	0.90*** [0.86,0.94]		1.00 [0.95,1.06]
**Asthma and chronic lung disease**																		
	**Criterion-based measure**			**Self-reported diagnoses**			**Criterion-based measure**			**Self-reported diagnoses**			**Criterion-based measure**			**Self-reported diagnoses**		
	**Model 1**	**Model 2**	**Model 3**	**Model 1**	**Model 2**	**Model 3**	**Model 1**	**Model 2**	**Model 3**	**Model 1**	**Model 2**	**Model 3**	**Model 1**	**Model 2**	**Model 3**	**Model 1**	**Model 2**	**Model 3**
Male	1.71 [0.66,4.40]	1.97 [0.78,4.96]	0.92 [0.26,3.25]	0.56 [0.29,1.11]	0.58 [0.30,1.14]	0.50 [0.22,1.13]	3.49 [0.37,32.8]	3.01 [0.39,23.5]	3.84 [0.41,36.0]	0.31* [0.11,0.84]	0.30* [0.10,0.89]	0.30* [0.10,0.88]	0.48 [0.16,1.45]	0.54 [0.14,2.04]	0.50 [0.12,2.18]	0.97 [0.45,2.12]	1.04 [0.47,2.30]	0.88 [0.37,2.11]
Asset	0.76 [0.41,1.40]	1.03 [0.47,2.23]		0.97 [0.42,2.24]	0.95 [0.39,2.28]		6.51 [0.92,45.8]	6.19 [0.81,47.5]		1.42 [0.45,4.46]	1.32 [0.38,4.66]		0.45 [0.14,1.44]	0.80 [0.11,5.55]		0.85 [0.39,1.84]	1.05 [0.43,2.56]	
Education	0.71*** [0.64,0.78]		0.78** [0.66,0.93]	0.86** [0.76,0.96]		0.91 [0.81,1.01]	0.99 [0.95,1.04]		0.96 [0.86,1.08]	1.12 [0.94,1.34]		1.14 [0.98,1.33]	0.72*** [0.66,0.79]		0.90* [0.82,0.99]	0.85*** [0.79,0.91]		0.91 [0.83,1.00]

**Visual impairment**	**Criterion-based measure**			**Self-reported diagnoses**			**Criterion-based measure**			**Self-reported diagnoses**			**Criterion-based measure**			**Self-reported diagnoses**		
	**Model 1**	**Model 2**	**Model 3**	**Model 1**	**Model 2**	**Model 3**	**Model 1**	**Model 2**	**Model 3**	**Model 1**	**Model 2**	**Model 3**	**Model 1**	**Model 2**	**Model 3**	**Model 1**	**Model 2**	**Model 3**
Male	1.42 [0.78,2.57]	1.44 [0.80,2.59]	1.07 [0.53,2.16]	0.58 [0.20,1.70]	0.61 [0.20,1.88]	0.39 [0.14,1.08]	0.91 [0.50,1.65]	0.97 [0.55,1.70]	0.91 [0.50,1.67]	0.87 [0.41,1.86]	0.92 [0.39,2.16]	0.91 [0.38,2.20]	1.04 [0.74,1.47]	1.20 [0.83,1.73]	1.32 [0.91,1.89]	1.01 [0.64,1.57]	1.23 [0.74,2.04]	1.01 [0.62,1.65]
Asset	1.03 [0.58,1.84]	1.02 [0.53,1.98]		0.63 [0.14,2.94]	0.57 [0.11,3.03]		0.67 [0.30,1.53]	0.84 [0.37,1.92]		0.79 [0.21,2.99]	0.82 [0.19,3.52]		0.67* [0.46,0.97]	0.92 [0.59,1.45]		0.56* [0.31,0.99]	0.89 [0.44,1.81]	
Education	1.06 [0.94,1.20]		1.09 [0.96,1.23]	0.85* [0.74,0.98]		0.91 [0.75,1.10]	0.90 [0.81,1.01]		0.98 [0.89,1.07]	0.92 [0.79,1.07]		0.99 [0.89,1.10]	0.87*** [0.82,0.92]		0.93* [0.89,0.98]	0.90*** [0.84,0.96]		1.03 [0.96,1.10]

**Depression**	**Symptom-based measure**			**Self-reported diagnoses**			**Symptom-based measure**			**Self-reported diagnoses**			**Symptom-based measure**			**Self-reported diagnoses**		
	**Model 1**	**Model 2**	**Model 3**	**Model 1**	**Model 2**	**Model 3**	**Model 1**	**Model 2**	**Model 3**	**Model 1**	**Model 2**	**Model 3**	**Model 1**	**Model 2**	**Model 3**	**Model 1**	**Model 2**	**Model 3**
Male	0.098*** [0.03,0.29]	0.17*** [0.068,0.43]	0.16** [0.048,0.55]	0.086*** [0.03,0.25]	0.085*** [0.03,0.25]	0.078*** [0.02,0.26]	0.16** [0.05,0.53]	0.18** [0.060,0.55]	0.17** [0.050,0.58]	0.054*** [0.03,0.11]	0.051*** [0.03,0.10]	0.048*** [0.02,0.098]	0.75 [0.33,1.68]	0.87 [0.38,2.01]	0.65 [0.25,1.67]	0.53 [0.22,1.28]	0.54 [0.22,1.31]	0.56 [0.23,1.34]
Asset	0.67* [0.46,1.00]	0.56 [0.19,1.61]		1.33 [0.56,3.15]	1.04 [0.38,2.86]		1.48 [0.54,4.07]	1.53 [0.46,5.05]		1.22 [0.63,2.37]	1.43 [0.63,3.28]		0.71 [0.35,1.43]	0.86 [0.39,1.87]		1.38 [0.58,3.28]	1.45 [0.61,3.48]	
Education	0.91* [0.84,0.98]		0.87* [0.76,0.99]	0.98 [0.90,1.07]		1.00 [0.91,1.10]	1.00 [0.86,1.17]		1.03 [0.87,1.21]	0.97 [0.88,1.07]		1.00 [0.91,1.10]	1.01 [0.94,1.08]		1.04 [0.96,1.12]	0.99 [0.90,1.07]		0.97 [0.86,1.08]

Notes: i) 95% confidence intervals in brackets; ii) * $p < 0.05$, ** $p < 0.01$, *** $p < 0.001$; iii) dependent variable is symptom-based/criterion-based measure and self-reported diagnoses of each NCD, and is defined as 1 if the respondent had the NCD, and 0 if otherwise; iv) Male is dichotomous variable with female as reference category, asset is defined as the asset index of the household, and education is defined as years of education; v) Model 1 estimated unadjusted odds ratio by regressing each independent variable on NCD prevalence separately; Model 2 is adjusted for rural-urban, age, gender and asset, where Model 3 is adjusted for rural-urban, age, gender and education. Model 2 and 3 of symptom-based/criterion-based measures of each was also adjusted with a dichotomous variable of whether the respondent has taken medication/treatment or not.

Web Appendix Table 7: Summary of the socioeconomic pattern of prevalence between self-reported diagnoses and symptoms-based/criterion-based measures of specific NCDs in China, Ghana, India, Mexico, Russia, and South Africa

	Angina		Hypertension		Asthma and lung diseases		Visual impairments		Depression	
	Symptoms-based/criterion-based measures	Self-reported diagnoses	Symptoms-based/criterion-based measures	Self-reported diagnoses	Symptoms-based/criterion-based measures	Self-reported diagnoses	Symptoms-based/criterion-based measures	Self-reported diagnoses	Symptoms-based/criterion-based measures	Self-reported diagnoses
Concentration of NCD among **poor**	Ghana, India, China, Mexico, Russia	-	China, South Africa, Mexico, Russia	-	India, China, South Africa, Russia	Ghana, India, China, Mexico	Ghana, India, China, Mexico, Russia	Mexico, Russia	India, China, South Africa, Russia	-
Concentration of NCD among **less educated**	Ghana, India, South Africa, Mexico, Russia	South Africa	Ghana, India, China, South Africa, Russia	Russia	India, South Africa , Russia	Ghana, China, South Africa	Ghana, India, Mexico, Russia	South Africa	India, China, South Africa	China
Concentration of NCD among **rich**	South Africa*	Ghana, India, China, South Africa, Mexico	Ghana*, India*	Ghana, India, China, South Africa, Mexico, Russia	Ghana, Mexico	South Africa, Russia	South Africa*	Ghana, India, China, South Africa	Ghana*, Mexico	Ghana, India, China, South Africa, Mexico, Russia
Concentration of NCD among **more educated**	China*	Ghana, India, China, Mexico, Russia	Mexico	Ghana, India, China, South Africa	Ghana, China, South Africa, Mexico	India, Mexico	China*, South Africa	Ghana, India, China, Mexico, Russia	Ghana*, Mexico*, Russia	Ghana, India, South Africa, Mexico, Russia

** With trends in attenuation (turned to less positive) in the magnitude of inequality by detecting more prevalence cases from lower SES*

Web Appendix Table 8: Age-adjusted prevalence rate (in %, with 95% CI) of NCDs among men and women, measured through self-reported diagnoses and symptoms-based/criterion-based measure, among adult population in China, Ghana, India, Mexico, Russia, and South Africa

	Angina		Hypertension		Asthma and chronic lung diseases		Visual impairment		Depression	
	Symptoms-based measure	Self-reported diagnoses	Criterion-based measure	Self-reported diagnoses	Criterion-based measure	Self-reported diagnoses	Criterion-based measure	Self-reported diagnoses	Symptoms-based measure	Self-reported diagnoses
**Ghana**										
Men	2.7[1.7; 3.7]	0.9[0.6; 1.3]	43.8[40.1; 47.5]	6.2[4.2; 8.1]	1.5[0.9; 2.1]	1.9[1.0; 2.9]	15.3[12.8; 17.8]	1.6[1.1; 2.1]	3.8[2.4; 5.3]	0.9[0.1; 1.6]
Women	5.0[3.6; 6.4]	2.5[1.2; 3.9]	42.9[38.8; 47.0]	9.4[7.1; 11.6]	1.7[0.5; 2.8]	2.0[1.1; 2.9]	12.5[10.3; 14.7]	2.5[1.6; 3.3]	4.7[3.3; 6.2]	0.6[0.4; 0.8]
All Sample	3.9[3.0;4.8]	1.7[1.0;2.3]	42.2[39.4;45.0]	7.4[6.0;8.8]	1.4[0.8;2.1]	2.2[1.5;3.0]	13.9[12.2;15.7]	2.3[1.6;2.9]	4.2[3.2;5.2]	0.8[0.3;1.2]
**India**										
Men	6.2[4.8; 7.5]	3.2[2.4; 4.0]	19.3[17.3; 21.4]	7.0[5.8; 8.2]	5.0[3.9; 6.1]	4.5[3.6; 5.5]	14.3[13.0; 15.7]	5.7[4.9; 6.5]	7.5[6.1; 8.8]	4.1[3.2; 5.1]
Women	8.2[7.3; 9.1]	3.0[2.5; 3.6]	19.5[18.1; 20.8]	12.4[11.3; 13.5]	5.6[4.8; 6.4]	3.2[2.6; 3.9]	19.6[18.4; 20.8]	6.5[5.9; 7.2]	10.1[9.1; 11.0]	2.6[1.9; 3.2]
All Sample	7.0[6.2;7.8]	3.1[2.6;3.6]	19.1[17.9;20.3]	9.4[8.6;10.2]	5.2[4.6;5.9]	5.8[5.1;6.6]	16.7[15.8;17.7]	6.1[5.5;6.6]	8.5[7.7;9.3]	3.4[2.8;3.9]
**China**										
Men	1.0[0.6; 1.4]	3.2[2.1; 4.4]	41.1[38.1; 44.1]	10.4[8.9; 11.8]	0.9[0.6; 1.2]	1.5[0.8; 2.2]	13.2[11.4; 15.1]	2.3[1.4; 3.1]	1.1[0.4; 1.8]	0.3[-0.1; 0.7]
Women	1.9[1.2; 2.6]	4.3[3.5; 5.2]	35.0[32.5; 37.4]	12.2[10.8; 13.7]	2.0[1.3; 2.7]	0.8[0.5; 1.1]	16.7[14.8; 18.7]	3.0[2.6; 3.3]	0.9[0.5; 1.3]	0.4[0.0; 0.9]
All Sample	1.4[1.0;1.8]	3.8[3.1;4.5]	38.0[36.0;40.0]	11.3[10.2;12.4]	1.4[1.1;1.7]	4.2[3.5;4.9]	14.8[13.5;16.2]	2.6[2.2;3.1]	1.0[0.6;1.5]	0.4[0.1;0.7]
**South Africa**										
Men	0.7[0.4; 1.0]	1.4[0.7; 2.1]	55.4[48.5; 62.3]	7.4[6.1; 8.6]	1.0[0.4; 1.6]	1.8[1.0; 2.5]	22.4[17.3; 27.5]	1.8[0.5; 3.1]	0.9[0.5; 1.4]	0.9[0.4; 1.4]
Women	2.2[1.1; 3.3]	3.9[1.2; 6.7]	46.7[41.3; 52.1]	11.8[9.6; 14.0]	0.6[0.3; 0.9]	2.8[1.6; 4.1]	18.4[14.8; 21.9]	3.1[1.0; 5.2]	7.9[4.1; 11.6]	9.1[4.9; 13.2]
All Sample	1.6[0.8;2.3]	2.7[1.4;3.9]	49.1[43.5;54.7]	9.9[8.5;11.4]	0.8[0.5;1.2]	4.2[2.2;6.2]	21.6[17.5;25.6]	2.2[1.0;3.3]	4.2[1.3;7.0]	4.6[1.6;7.5]
**Mexico**										
Men	4.0[2.0; 5.9]	0.5[0.2; 0.7]	33.3[27.1; 39.4]	10.0[7.7; 12.4]	3.8[-0.6; 8.3]	0.7[0.2; 1.2]	22.9[18.7; 27.2]	4.1[1.9; 6.3]	1.8[0.3; 3.3]	1.4[0.7; 2.0]

Women	8.2[5.7; 10.8]	1.9[0.5; 3.3]	27.3[22.6; 32.1]	15.7[12.5; 18.9]	0.5[-0.1; 1.0]	2.0[1.1; 2.9]	24.5[20.5; 28.6]	4.0[2.6; 5.4]	9.7[6.9; 12.6]	18.4[14.7; 22.1]
All Sample	6.5[4.5;8.5]	1.1[0.4;1.8]	27.7[23.5;31.8]	13.2[11.0;15.4]	1.2[-0.6;3.1]	6.6[2.4;10.7]	24.3[20.9;27.7]	4.0[2.8;5.1]	6.3[4.3;8.3]	10.1[7.8;12.5]
**Russia**										
Men	14.0[10.8;17.2]	23.1[19.7; 26.5]	42.2[38.2; 46.2]	31.7[27.6; 35.8]	0.3[0.0; 0.6]	4.7[2.6; 6.8]	23.7[20.6; 26.9]	10.3[7.9; 12.8]	4.4[2.1; 6.7]	2.4[0.9; 3.9]
Women	16.7[14.5;18.9]	24.8[22.0; 27.7]	41.8[38.8; 44.9]	41.0[37.9; 44.2]	0.3[0.1; 0.5]	5.5[3.5; 7.5]	23.1[20.4; 25.8]	9.4[7.4; 11.4]	5.2[3.6; 6.9]	4.7[2.9; 6.6]
All Sample	15.7[13.5;17.9]	23.8[21.2;26.3]	41.9[39.1;44.6]	36.7[33.9;39.4]	0.3[0.1;0.4]	15.0[12.8;17.2]	24.3[21.9;26.8]	9.6[8.1;11.2]	4.8[3.3;6.3]	3.9[2.4;5.3]

Source: WHO SAGE survey, 2007-10

Web Appendix Table 9: Age-adjusted wealth-related and education-related concentration index (in %, with 95% CI) of disease, measured through self-reported diagnoses and symptoms-based/criterion-based measures, among adult men and women in China, Ghana, India, Mexico, Russia, and South Africa

	**Wealth-related concentration index**				**Education-related concentration index**			
	**Men**		**Women**		**Men**		**Women**	
	Symptoms-based/criterion-based measure	Self-reported diagnoses	Symptoms-based/criterion-based measure	Self-reported diagnoses	Symptoms-based/criterion-based measure	Self-reported diagnoses	Symptoms-based/criterion-based measure	Self-reported diagnoses
**Angina**								
Ghana	-0.20[-0.22;-0.18]	0.09[0.07; 0.10]	-0.19[-0.22;-0.17]	0.06[0.03; 0.10]	0.02[0.00;0.03]	0.06[0.02; 0.10]	-0.26[-0.28;-0.24]	0.54[0.50; 0.58]
India	-0.24[-0.26;-0.23]	0.05[0.04; 0.05]	-0.10[-0.11;-0.10]	-0.02[-0.02; -0.02]	-0.11[-0.12;-0.10]	0.00[-0.01; 0.01]	0.00[0.00;0.00]	-0.03[-0.03; -0.03]
China	-0.06[-0.06;-0.05]	0.16[0.15; 0.16]	-0.11[-0.12;-0.11]	0.00[0.00; 0.01]	0.01[-0.01;0.03]	0.03[0.01; 0.05]	0.03[0.02;0.04]	0.19[0.18; 0.20]
South Africa	-0.32[-0.37;-0.26]	0.42[0.34; 0.50]	0.07[0.01;0.14]	0.37[0.30; 0.45]	-0.09[-0.14;-0.05]	-0.01[-0.03; 0.02]	-0.24[-0.30;-0.18]	-0.14[-0.21; -0.07]
Mexico	-0.48[-0.57;-0.39]	0.21[0.13; 0.28]	-0.10[-0.14;-0.05]	0.18[0.16; 0.21]	-0.22[-0.27;-0.17]	0.24[0.08; 0.39]	-0.01[-0.06;0.03]	0.04[-0.03; 0.11]
Russia	0.00[-0.01;0.01]	-0.01[-0.03; 0.00]	-0.03[-0.03;-0.02]	0.00[0.00; 0.00]	-0.12[-0.15;-0.09]	0.08[0.06; 0.09]	0.00[-0.01;0.01]	0.02[0.02; 0.03]
**Hypertension**								
Ghana	0.01[0.01;0.02]	0.45[0.42; 0.48]	0.05[0.04;0.06]	0.25[0.23; 0.28]	0.00[0.00;0.01]	0.12[0.11; 0.14]	-0.08[-0.09;-0.07]	0.10[0.09; 0.12]
India	0.06[0.05;0.06]	0.21[0.20; 0.23]	-0.01[-0.01;-0.01]	0.17[0.17; 0.18]	0.01[0.00;0.01]	0.17[0.16; 0.18]	-0.02[-0.02;-0.02]	0.02[0.01; 0.02]
China	0.00[0.00;0.00]	0.12[0.12; 0.13]	-0.04[-0.04;-0.03]	0.06[0.06; 0.06]	-0.02[-0.02;-0.01]	0.11[0.10; 0.12]	-0.07[-0.07;-0.07]	0.01[0.01; 0.01]
South Africa	0.02[0.01;0.04]	0.11[0.08; 0.15]	-0.04[-0.05;-0.03]	0.18[0.15; 0.21]	0.03[0.00;0.06]	-0.10[-0.15; -0.05]	-0.06[-0.08;-0.05]	0.05[0.00; 0.11]
Mexico	-0.01[-0.03;0.00]	0.18[0.13; 0.23]	-0.01[-0.01;0.00]	0.13[0.09; 0.16]	0.10[0.07;0.13]	0.25[0.18; 0.32]	0.10[0.07;0.13]	-0.13[-0.14; -0.11]
Russia	-0.11[-0.13;-0.10]	0.08[0.07; 0.08]	-0.02[-0.03;-0.02]	0.02[0.01; 0.02]	-0.01[-0.01;0.00]	0.02[0.01; 0.03]	-0.03[-0.04;-0.03]	-0.06[-0.07; -0.05]
**Asthma and chronic lung disease**								
Ghana	-0.11[-0.13;-0.09]	0.00[-0.02; 0.02]	0.22[0.19;0.25]	0.04[0.02; 0.06]	-0.19[-0.21;-0.17]	-0.05[-0.08; -0.03]	0.40[0.36;0.43]	-0.30[-0.33; -0.28]
India	-0.06[-0.06;-0.05]	0.02[0.01; 0.02]	-0.02[-0.03;-0.02]	0.00[-0.01; 0.00]	0.03[0.01;0.04]	0.06[0.05; 0.06]	0.04[0.03;0.04]	-0.02[-0.02; -0.02]
China	-0.27[-0.28;-0.26]	0.26[0.24; 0.28]	0.06[0.06;0.07]	-0.23[-0.24; -0.22]	0.07[0.04;0.09]	-0.15[-0.19; -0.11]	0.01[0.00;0.01]	0.03[0.01; 0.05]
South Africa	-0.08[-0.11;-0.05]	-0.22[-0.29; -0.15]	-0.15[-0.22;-0.08]	0.06[0.03; 0.08]	0.13[-0.17;0.43]	-0.13[-0.17; -0.09]	-0.24[-0.34;-0.15]	-0.17[-0.24; -0.10]
Mexico	0.77[0.65;0.89]	-0.28[-0.35; -0.21]	-0.22[-0.28;-0.16]	0.17[0.13; 0.21]	0.12[-0.03;0.27]	0.09[-0.04; 0.21]	0.26[0.20;0.33]	0.18[0.12; 0.24]
Russia	-0.22[-0.26;-0.19]	-0.16[-0.19; -0.12]	-0.11[-0.15;-0.07]	0.09[0.07; 0.12]	-0.43[-0.49;-0.38]	0.15[0.09; 0.21]	-0.30[-0.34;-0.26]	-0.10[-0.14; -0.06]
**Visual impairment**								

Ghana	-0.06[-0.06;-0.05]	-0.03[-0.04; -0.03]	-0.07[-0.08;-0.06]	0.41[0.39; 0.44]	-0.06[-0.07;-0.05]	0.09[0.07; 0.11]	0.00[-0.01;0.02]	0.38[0.36; 0.41]
India	-0.05[-0.06;-0.05]	0.07[0.06; 0.07]	-0.06[-0.07;-0.06]	0.07[0.07; 0.08]	-0.05[-0.05;-0.04]	0.07[0.07; 0.07]	-0.05[-0.06;-0.05]	-0.05[-0.05; -0.05]
China	-0.04[-0.04;-0.03]	0.04[0.02; 0.06]	0.01[0.00;0.01]	0.05[0.05; 0.05]	0.14[0.12;0.16]	0.20[0.18; 0.22]	0.08[0.07;0.09]	0.08[0.07; 0.09]
South Africa	-0.09[-0.10;-0.07]	-0.28[-0.36; -0.21]	0.18[0.14;0.23]	0.02[-0.03; 0.07]	0.00[-0.07;0.06]	-0.05[-0.09; -0.01]	0.08[0.06;0.11]	-0.27[-0.31; -0.23]
Mexico	-0.17[-0.24;-0.10]	-0.33[-0.41; -0.25]	-0.06[-0.08;-0.05]	0.10[0.07; 0.14]	-0.12[-0.17;-0.08]	-0.02[-0.10; 0.07]	-0.04[-0.06;-0.02]	0.16[0.11; 0.21]
Russia	-0.05[-0.07;-0.03]	-0.12[-0.13; -0.11]	-0.03[-0.04;-0.02]	-0.02[-0.03; -0.02]	-0.09[-0.10;-0.07]	0.24[0.22; 0.27]	0.01[0.00;0.02]	-0.18[-0.20; -0.17]
**Depression**								
Ghana	0.12[0.10;0.15]	0.35[0.31; 0.39]	-0.05[-0.06;-0.04]	0.04[0.03; 0.05]	-0.05[-0.08;-0.03]	0.14[0.10; 0.17]	0.08[0.07;0.08]	-0.22[-0.24; -0.20]
India	-0.18[-0.19;-0.17]	0.11[0.10; 0.12]	-0.09[-0.09;-0.08]	-0.02[-0.03; -0.02]	-0.10[-0.11;-0.09]	-0.02[-0.03; -0.01]	0.05[0.04;0.05]	-0.06[-0.07; -0.06]
China	0.02[0.00;0.05]	0.54[0.51; 0.56]	-0.28[-0.30;-0.27]	0.03[0.01; 0.05]	0.11[0.09;0.14]	0.40[0.37; 0.43]	-0.03[-0.05;-0.02]	-0.32[-0.34; -0.31]
South Africa	0.06[-0.02;0.15]	0.28[0.20; 0.36]	-0.24[-0.34;-0.15]	-0.01[-0.07; 0.05]	-0.07[-0.12;-0.03]	0.03[-0.02; 0.08]	-0.24[-0.31;-0.17]	0.05[-0.03; 0.13]
Mexico	0.36[0.30;0.42]	-0.09[-0.15; -0.04]	-0.02[-0.07;0.04]	0.08[0.05; 0.12]	0.44[0.35;0.54]	-0.05[-0.11; 0.00]	0.05[0.02;0.08]	0.13[0.09; 0.18]
Russia	0.02[-0.04;0.07]	0.29[0.26; 0.33]	0.01[0.00;0.03]	0.07[0.06; 0.08]	0.22[0.16;0.28]	-0.22[-0.26; -0.19]	0.08[0.07;0.09]	0.18[0.15; 0.20]

Notes: i. Source: WHO SAGE survey, 2007-10; ii. Negative values indicate concentration of disease among lower SES, and positive values indicate concentration among higher SES. The symptom-based/criterion-based measures of each NCD was adjusted with age, and a dichotomous variable of whether the respondent has taken medication/treatment or not.

Web Appendix Figure 1: Age-adjusted prevalence of symptoms-based/criterion-based measures of diseases among adult population in China, Ghana, India, Mexico, Russia, and South Africa, by wealth quintiles

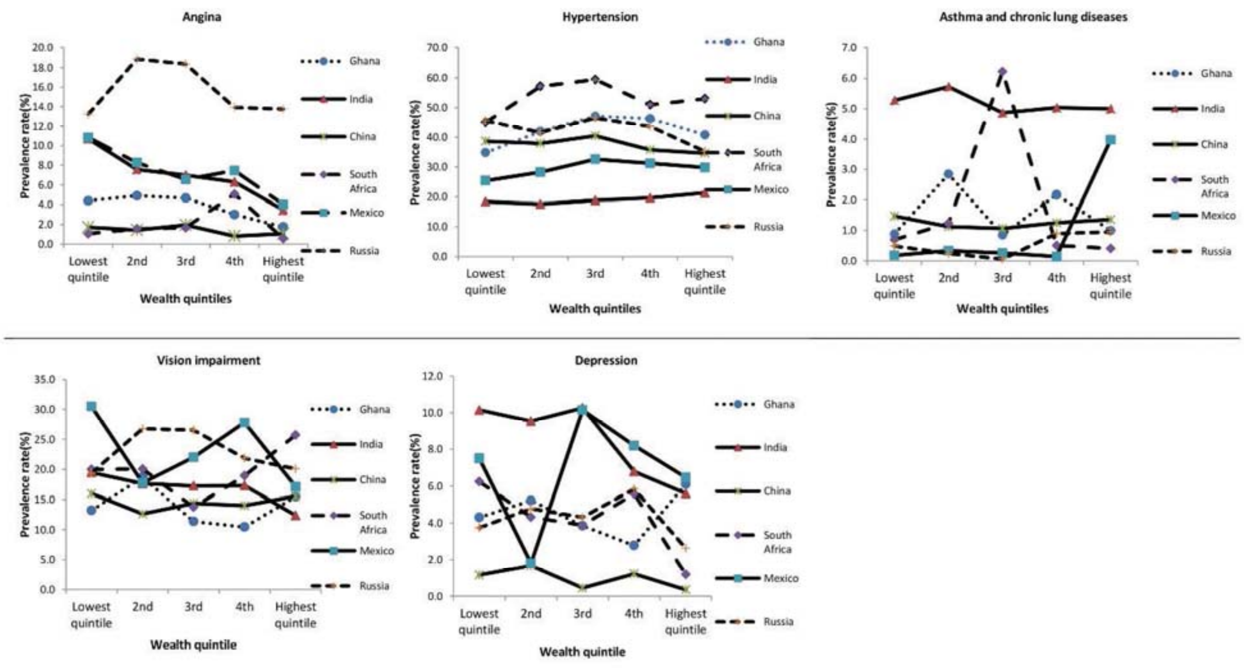

Source: WHO SAGE survey, 2007-10
